# Dapagliflozin reduces the vulnerability of rats with pulmonary arterial hypertension-induced right heart failure to ventricular arrhythmia by restoring calcium handling

**DOI:** 10.1186/s12933-022-01614-5

**Published:** 2022-09-28

**Authors:** Jinchun Wu, Tao Liu, Shaobo Shi, Zhixing Fan, Roddy Hiram, Feng Xiong, Bo Cui, Xiaoling Su, Rong Chang, Wei Zhang, Min Yan, Yanhong Tang, He Huang, Gang Wu, Congxin Huang

**Affiliations:** 1 Department of Cardiology, Renmin Hospital of Wuhan University, No. 238 Jiefang Road, Wuhan, 430060 People’s Republic of China; 2 Cardiovascular Research Institute, Wuhan University, 238 Jiefang Road, Wuhan, 430060 People’s Republic of China; 3 Hubei Key Laboratory of Cardiology, 238 Jiefang Road, Wuhan, 430060 People’s Republic of China; 4 Department of Cardiology, Qinghai Provincial People’s Hospital, No.2 Gong He Road, Xining, 810007 People’s Republic of China; 5 Department of Medicine, Faculty of Medicine, Montreal Heart Institute (MHI), Université de Montréal, Montreal, QC Canada; 6 Department of Cardiology, Shenzhen Longhua District Central Hospital, The Affiliated Central Hospital of Shenzhen Longhua District, Guangdong Medical University, No. 187 Guanlan Road, Longhua District, Shenzhen, 518109 China

**Keywords:** Dapagliflozin, Ventricular arrhythmias, Calcium handling, Right heart failure, Pulmonary arterial hypertension, Monocrotaline

## Abstract

**Background:**

Malignant ventricular arrhythmia (VA) is a major contributor to sudden cardiac death (SCD) in patients with pulmonary arterial hypertension (PAH)-induced right heart failure (RHF). Recently, dapagliflozin (DAPA), a sodium/glucose cotransporter-2 inhibitor (SGLT2i), has been found to exhibit cardioprotective effects in patients with left ventricular systolic dysfunction. In this study, we examined the effects of DAPA on VA vulnerability in a rat model of PAH-induced RHF.

**Methods:**

Rats randomly received monocrotaline (MCT, 60 mg/kg) or vehicle via a single intraperitoneal injection. A day later, MCT-injected rats were randomly treated with placebo, low-dose DAPA (1 mg/kg/day), or high-dose (3 mg/kg/day) DAPA orally for 35 days. Echocardiographic analysis, haemodynamic experiments, and histological assessments were subsequently performed to confirm the presence of PAH-induced RHF. Right ventricle (RV) expression of calcium (Ca^2+^) handling proteins were detected via Western blotting. RV expression of connexin 43 (Cx43) was determined via immunohistochemical staining. An optical mapping study was performed to assess the electrophysiological characteristics in isolated hearts. Cellular Ca^2+^ imaging from RV cardiomyocytes (RVCMs) was recorded using Fura-2 AM or Fluo-4 AM.

**Results:**

High-dose DAPA treatment attenuated RV structural remodelling, improved RV function, alleviated Cx43 remodelling, increased the conduction velocity, restored the expression of key Ca^2+^ handling proteins, increased the threshold for Ca^2+^ and action potential duration (APD) alternans, decreased susceptibility to spatially discordant APD alternans and spontaneous Ca^2+^ events, promoted cellular Ca^2+^ handling, and reduced VA vulnerability in PAH-induced RHF rats. Low-dose DAPA treatment also showed antiarrhythmic effects in hearts with PAH-induced RHF, although with a lower level of efficacy.

**Conclusion:**

DAPA administration reduced VA vulnerability in rats with PAH-induced RHF by improving RVCM Ca^2+^ handling.

**Supplementary Information:**

The online version contains supplementary material available at 10.1186/s12933-022-01614-5.

## Introduction

Pulmonary arterial hypertension (PAH) is a progressive and severe disease that is characterised by pathologic vascular remodelling that leads to right heart failure (RHF) or even sudden cardiac death (SCD) [1]. It is well known that malignant ventricular arrhythmia (VA) is a major cause of SCD [2]. Recent evidence has reported that the prevalence of VA in all forms of PAH is 24% [3]. Additionally, increased VA vulnerability has been demonstrated in animals with PHA-induced RHF [4, 5]. However, the exact mechanism by which VA arises in PAH-induced RHF is poorly understood. Evidence shows that Ca^2+^ handling abnormalities play a pivotal role in ventricular arrhythmogenesis in hearts with PAH-induced RHF [6, 7]. In this regard, mitigation of impaired Ca^2+^ handling could be a promising therapeutic approach for the prevention of VA after PAH-induced RHF.

Dapagliflozin (DAPA), a novel sodium/glucose cotransporter-2 inhibitor (SGLT2i), is clinically used to reduce blood glucose by blocking the reabsorption of glucose and sodium from the proximal tubules [8]. Recent studies have shown that DAPA exerts cardioprotective effects [9], for example, it exhibits antiarrhythmic effects and improves cardiac function in both animal and human hearts [10–12]. However, the effects of DAPA on right ventricle (RV) function and VA secondary to PAH-induced RHF remain unknown. A previous study has reported that SGLT2i treatment can reduce the activity of calcium/calmodulin-dependent protein kinase II (CaMKII) and improve Ca^2+^ handling in human and murine ventricular cardiomyocytes (CMs) [13]. Thus, we aimed to determine whether DAPA could decrease the vulnerability of rats with PAH-induced RHF to VA by restoring Ca^2+^ homeostasis.

## Materials and methods

### Experimental animals

All animal procedures followed the guidelines of the American Veterinary Medical Association (AVMA) for the Euthanasia of Animals (2020) and were approved by the Animal Ethics Committee of Renmin Hospital of Wuhan University, China (Number: 20201211). Adult male Sprague‒Dawley (SD) rats (220 to 280 g) were obtained from the Animal Centre of Renmin Hospital of Wuhan University. After 7 days of acclimatisation, the rats were randomly divided into the following 4 groups: the control (CTL) group, monocrotaline (MCT) group, MCT plus low-dose DAPA (MCT + LD) group, and MCT plus high-dose DAPA (MCT + HD) group. Following the methodology of a previous study [14], RHF was induced via a single intraperitoneal injection of MCT (60 mg/kg, dissolved in absolute ethanol, Absin, Shanghai, China). The rats were intraperitoneally injected with ethanol (1 mL/kg) in the CTL group. One day later, MCT-injected rats were randomly treated with placebo, low-dose DAPA (1 mg/kg/day), or high-dose DAPA (3 mg/kg/day) via oral gavage daily for 35 days. All rats were raised in a standard specific pathogen-free (SPF) environment (12 h light/12 h dark) where they could freely obtain food and water. The primary indication of RHF was more than 10 g of weight loss daily over consecutive days accompanied by other signs, including anorexia, dyspnoea, cold extremities, piloerection, and lethargy [15, 16].

### Echocardiographic analysis

Rats (n = 5 per group) were subsequently anaesthetised (isoflurane, 2%). Echocardiographic analysis was performed using a high-frequency single crystal probe colour ultrasound system (VINNO Technology, Suzhou, China). The RV end-diastolic middle width (RV width), RV end-diastolic length (RV length), pulmonary arterial diameter (PAD), pulmonary arterial acceleration time (PAAT), tricuspid annular plane systolic excursion (TAPSE), RV fractional area change (RVFAC), left ventricular end-systolic volume (LVESV), left ventricular end-diastolic volume (LVEDV), cardiac output (CO), and left ventricular ejection fraction (LVEF) were obtained via echocardiographic data measurement or calculation as previously described [17].

### Haemodynamic experiments and blood sampling

Rats (n = 8 per group) were anaesthetised intraperitoneally with pentobarbital sodium (40 mg/kg) and heparinised with heparin sodium (400 U). As previously described [18, 19], measurement of pulmonary arterial pressure (PAP) and right ventricular pressure (RVP) was performed in rat hearts by using a heparin-saline fluid-filled polyethylene (PE) catheter. Briefly, the right jugular vein was separated, and a 2F PE catheter transducer was then inserted into the pulmonary artery (PA) through the right atrium and the RV. After stabilisation for 10 min, the heart rate and pressure signal were monitored and analysed using a Power Lab amplifier system and software (Lab Chart 8.0, AD Instruments, Australia), respectively. The RVP and PAP were measured for 5 consecutive cardiac cycles, and then the means of these parameters were calculated. Subsequently, the blood samples (approximately 2 mL) were then collected from the right carotid artery and allowed to be coagulated for 1 h at room temperature. They were centrifuged for 5 min at 5,000 rpm to prepare the serum. Then, the serum N-terminal pro-brain natriuretic peptide (NT-pro-BNP) level was detected using the Rat NT-pro-BNP ELISA Kit (ELK Biotechnology. Ltd, Wuhan). The rats were then cervically dislocated, and the hearts were divided into the RV, left ventricle (LV), and interventricular septum (IVS). The Fulton index was calculated as a weight ratio, RV/(LV + IVS), to evaluate RV hypertrophy, as previously described [6].

### Histological and morphological analysis

RV-free wall tissues were collected and fixed in 4% paraformaldehyde for 24 h, embedded in paraffin, and sectioned into 5 µm-thick sections. Then, the sections were subjected to hematoxylin and eosin (H and E), wheat germ agglutinin (WGA) immunofluorescence, and Masson’s trichrome staining. The H and E (× 400) and Masson’s images (× 200) were observed using CaseViewer 2.4 software (3DHISTECH, Hungary), and Masson’s images were analysed by calculating the average collagen volume fraction (CVF) using Image-Pro Plus 6.0 software (Media Cybernetics, Inc). The WGA-stained images (× 400) were recorded using an inverted fluorescence microscope (IX51, Olympus, Japan) and were quantified by cardiomyocyte cross-sectional area using Image-Pro Plus 6.0 software [20, 21]. The remodelling of PA and apoptosis of right ventricular CMs (RVCMs ) were investigated in this study (Additional file 8: Supplemental methods).

### Detection of CD31 and Cx43 expression in the RV by immunohistochemical staining

As previously described [22, 23], immunohistochemical staining of cluster of differentiation 31 (CD31) is the standard method of quantifying capillary vasculature. Briefly, the RV sections were stained with a rabbit polyclonal antibody against CD31 (GB11063-3, 1:400, Service, China), then incubated with secondary antibody according to the manufacturer’s instructions. CD31-positive areas were classified as those with any brown-stained individual endothelial cells or clusters of endothelial cells, which were considered capillaries. The capillaries were counted in 5 different microscopic fields of each section, and the capillary density was calculated as the average positive number of the vessels in one section. All images (× 400) were observed using CaseViewer 2.4 software and analysed using Image-Pro Plus 6.0 software. As previously described [24], the expression of connexin 43 (Cx43) in the RV was detected with a Cx43 rabbit polyclonal antibody (GB12234, 1:300, Servicebio, China), and images (× 400) were observed using CaseViewer 2.4 software and analysed using Halo v3.0.311.314 software (Indica Labs, USA). The percentage of average positive expression area (PEA) of Cx43 represented Cx43 expression.

### Western blot analysis

RV-free wall tissue was collected and stored in tubes at – 80 ℃. The protein expression of total ryanodine receptor 2 (t-RyR2) (Affinity, AF0015, 1:500), Ser2814-phosphorylated RyR2 (p-RyR2) (Badrilla, A010-31AP, 1:300), sarco-/endoplasmic reticulum Ca^2+^ ATPase 2a (SERCA2a) (Abcam, Ab150435, 1:1000), phospholamban (PLB) (Abcam, Ab85146, 1:1000), total CaMKII (t-CaMKII) (Abcam, Ab134041, 1:1000), Thr287-phosphorylated CaMKII (p-CaMKII) (Thermo Fisher, PA5-37833, 1:500), Cav 1.2 (Abcam, Ab58552, 1:500) and Na^+^-Ca^2+^ exchanger (NCX) (Invitrogen, MA3-926, 1:500) was determined via Western blotting and was normalised to glyceraldehyde-3-phosphate dehydrogenase (GAPDH) (Abcam, ab181602, 1:10000) expression, as previously described [25, 26].

### Calcium imaging study

#### RVCM isolation

RVCMs were isolated from the RV as previously described [27]. Briefly, rats were intraperitoneally anaesthetised with pentobarbital sodium (40 mg/kg) that was heparinised with heparin sodium (400 U). After thoracotomy, the isolated heart was quickly removed and placed in a Langendorff system. The heart was retrogradely perfused at a constant flow with normal Tyrode’s solution (6–8 mL/min, 36–37 ℃). The normal Tyrode solution contained the following: NaCl (145.0 mmol/L), KCl (5.9 mmol/L), CaCl_2_ (1.1 mmol/L), MgCl_2_ (1.2 mmol/L), glucose (11.0 mmol/L), and HEPES (5.0 mmol/L). The pH of the solution was adjusted to 7.4 by adding NaOH [28]. Once heart contraction stabilised, the heart was perfused with Ca^2+^-free Tyrode’s solution for 15–20 min, followed by Ca^2+^-free Tyrode’s solution containing collagenase type II plus 0.1% bovine serum albumin for a total digestion time of 6–8 min. The digested tissue was carefully minced and agitated, and RVCMs were harvested. After isolation, the cells were kept in Ca^2+^ (0.2 mmol/L)-containing Tyrode’s solution for the subsequent Ca^2+^ imaging experiments, which were conducted within 5 h [26, 29].

#### Ca^2+^microfluorometry

RVCMs were incubated with Fura-2-AM (5 µM) for 15 to 20 min at room temperature. Subsequently, the RVCMs were superfused with normal Tyrode solution for 20 to 25 min to ensure complete de-esterification of intracellular Fura-2 AM. The RVCMs were alternately excited using 340/380 nm of light, and the emitted fluorescence was recorded at 510 nm. The ratio of fluorescence emitted at 510 nm in response to the two excitation wavelengths (ratio: 340/380) was calculated to provide an index of diastolic intracellular Ca^2+^ ([Ca^2+^]_i_) levels. Background fluorescence was subtracted before the 340/380 fluorescence ratio was obtained. Ca^2+^ transients (CaT) were elicited via electrical field stimulation at 1 Hz. The diastolic [Ca^2+^]_i_ level, the CaT amplitude and the CaT decay time constant were measured according to the methods that were previously described methods [21, 30].

Additionally, the Ca^2+^ content of the sarcoplasmic reticulum ([Ca^2+^]_SR_) was measured by rapidly adding 10 mmol of caffeine using a solution switching device after a train of 1 Hz electrical field stimulation. The amplitude of caffeine-induced CaT amplitude was calculated to assess [Ca^2+^]_SR_. Spontaneous Ca^2+^ events (SCaEs) were observed during the first 20 s after Ca^2+^ loading by 4 s periods of pacing at 5 Hz. A successful SCaE was defined as an unstimulated CaT that reached at least 20% of the steady-state signal at 5 Hz. The threshold of Ca^2+^ alternans was determined via the S1S1 protocol [31, 32]. Briefly, Ca^2+^ alternans was elicited during S1–S1 pacing with 5 s pulse trains separated by 10 s intervals to minimise pacing memory. Starting at 1 Hz, the pacing frequency was increased in 1-Hz steps until Ca^2+^ alternans or a 2:1 block was induced. Ca^2+^ alternans was quantified by subtracting the CaT amplitude from two consecutive beats. Ca^2+^ alternans was defined as the occurrence of interbeat CaT amplitude differences averaging more than 5% over six stimuli. The threshold for Ca^2+^ alternans was defined as the minimum pacing frequency at which the Ca^2+^ alternans occurred [33]. All measurements were obtained and analysed using IonWizard 6.5 software (IonOptix Corporation, Milton, MA, USA).

#### Confocal Ca^2+^imaging

According to our previous study [34], RVCMs were loaded with 5 µmol/L Fluo-4 AM for 30 min. The RVCMs were then placed on glass coverslips and superfused with 1.8 mmol/L Ca^2+^ containing Tyrode’s solution at 20–22 °C for 10 min. To assess spontaneous Ca^2+^ sparks (SCaSs), confocal Ca^2+^ imaging was performed using a confocal microscope (Leica, Wetzlar, Germany, equipped with a 63 × apochromatic oil objective) in line-scan mode. Fluo-4 AM was excited with a 488 nm argon laser, and emission fluorescence was collected at 505 nm.

RVCMs were field-stimulated at 1 Hz to obtain steady-state [Ca^2+^]_SR_. The last paced event was recorded along with the subsequent 2 − 3 s for SCaS recording. The resting Ca^2+^ sparks were automatically detected and characterised using custom-written algorithms in MATLAB software. Background-subtracted fluorescence emission signals (F) were normalised to baseline fluorescence (F_0_) by averaging 10 images. The [Ca^2+^]_i_ changes are presented as ΔF/F_0_ (where ΔF = F−F_0_) [32]. The parameters that were analysed included SCaS frequency, SCaS amplitude, SCaS width, SCaS duration to half-decay, SCaS mass, and SCaS-mediated leakage. We simply followed previously described approaches to analyse the aforementioned parameters [6].

### Optical mapping study

Rats (n = 10 per group) were anaesthetised and heparinised as previously described. After thoracotomy, the isolated hearts were quickly removed and placed in a Langendorff system. They were perfused retrogradely at a constant flow with Tyrode’s solution (6–8 mL/min, 36–37 ℃). After 20 min of stabilisation, the hearts were bolus-stained with a recirculating solution of 15 µM blebbistatin to suppress mechanical contraction, and then Di-4-ANEPPS (10 µM, 0.1 mL) was injected via the perfusate to detect voltage-fluorescence signals. Fluorescence signals were recorded at 2 kHz with a charge-coupled device focused on a region of approximately 8 × 8 mm in the RV free wall (high-speed charge-coupled device camera system, RedShirt Imaging). The hearts were electrically paced from the basal surface of the RV at a 300 ms cycle length (5 mA, 2 ms pulse width). Images were acquired at 500 frames per second for consistency and the optimal signal-to-noise ratio. The conduction velocity (CV) was analysed using a MATLAB^®^ custom-written algorithm, as previously described [35, 36].

In addition, APD alternans was induced following an S1–S1 pacing protocol, as previously described [32]. Briefly, starting at 300 ms, the basic cycle length (BCL) was shortened to 200 ms in 20-ms intervals and subsequently in 10 ms steps until VA was induced or until 1:1 capture failed. APD_80_ was calculated as the time from the maximal upstroke velocity to 80% repolarisation. The APD alternans was assessed by subtracting the APD_80_ for two consecutive beats when the alternate APD_80_ differed by > 5% over six beats [37]. The phase of alternans was termed positive for long-short APD sequences and negative for short-long APD sequences. To evaluate the spatial characteristics, alternans was classified as spatial concordant or discordant alternans (SCA or SDA), as in our previous study [32]. The threshold for APD alternans was defined as the maximum BCL that induced APD alternans. VA was defined as a series of 2-s consecutive or more premature ventricular contractions [38].

### Statistical analysis

All experimental data were analysed using IBM SPSS, version 25, or GraphPad Prism software, version 8.0.2. The coefficients of variation are presented as the mean ± SEM unless otherwise indicated. The *p* values were calculated using one-way ANOVA or Tukey's multiple comparisons test. To calculate the threshold values for the Ca^2+^/APD alternans, which did not follow a normal distribution, the median with interquartile range was used to represent the central tendency and was compared using the nonparametric Wilcoxon signed-rank test or the Kruskal–Wallis test, as needed. The incidences of SDA and VA were compared via the *χ*^2^ tests. Optical mapping, microfluorometry, and confocal microscopy imaging data were processed using custom-made analysis software written in MATLAB (version 7.11, MathWorks). *p* < 0.05 was considered to indicate statistical significance.

## Results

### Effects of DAPA on the structural and functional remodelling of the RV

**Fig. 1:**
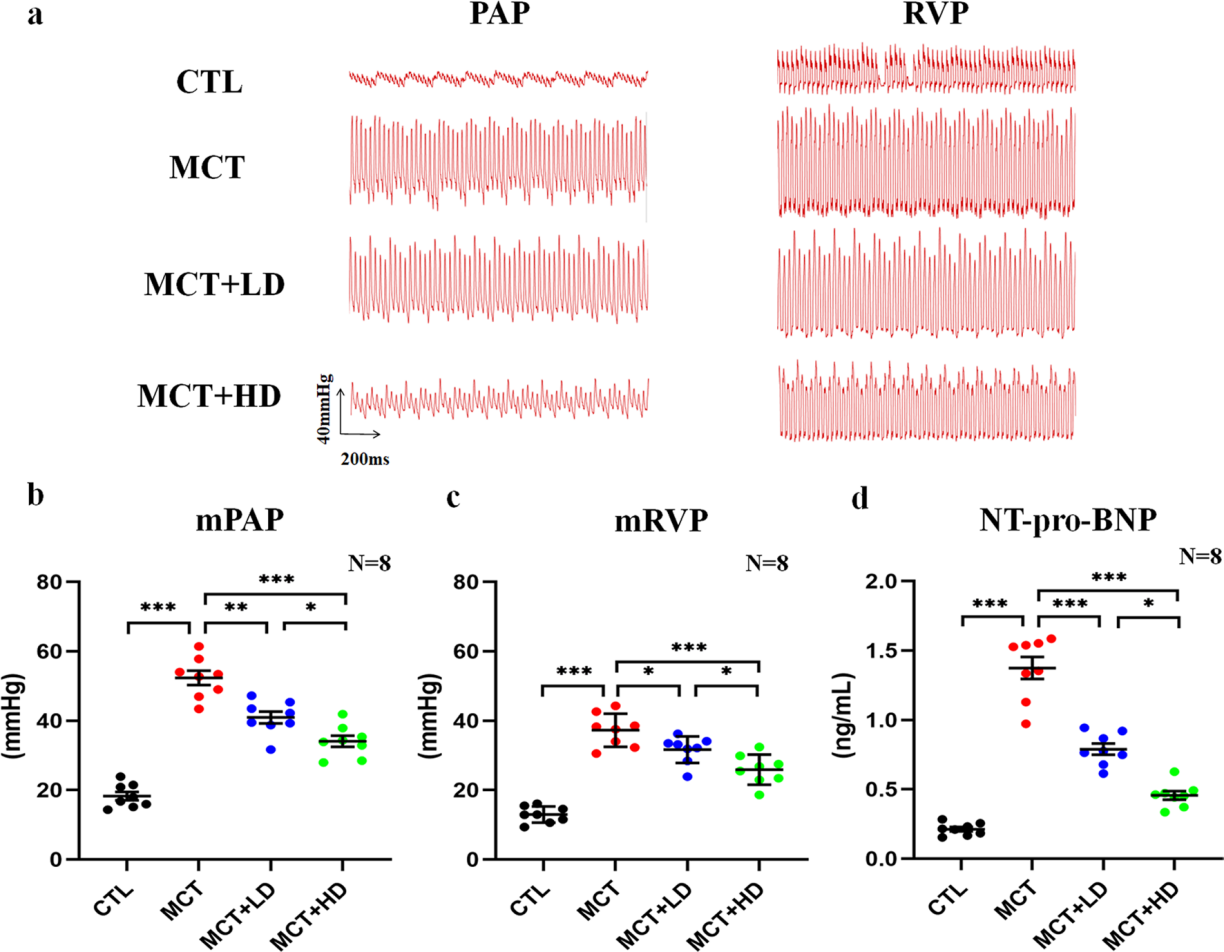
Measurement and analysis of PAP and RVP in the 4 groups of rats. **a** Representative PAP waves and RVP waves in the 4 groups of rats from haemodynamic experiments. **b** mPAP in the 4 groups of rats. **c** mRVP in the 4 groups of rats. **d** The NT-pro-BNP level in the 4 groups of rats. *mPAP* mean pulmonary arterial pressure, *mRVP* mean right ventricular pressure, *NT-pro-BNP* the serum N-terminal pro-Brain natriuretic peptide. N = 8 per group. The horizontal lines show the mean ± SEM. One-way ANOVA, ^*^p < 0.05, ^**^p < 0.01, ^***^p < 0.001

**Fig. 2:**
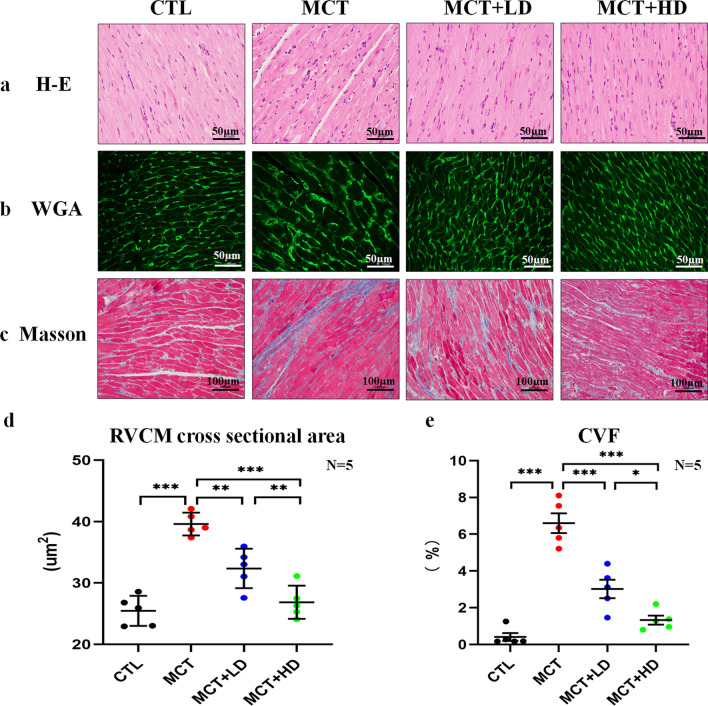
Histological analysis of RV tissue in the 4 groups of rats. **a** Representative image of the RV cryosections stained with H&E (× 400). Nuclei were stained dark purple, the cytoplasm was stained red, the scale bar is 50 µm. **b** Representative images of the RV WGA immunofluorescence detection (× 400), the scale bar is 50 µm. **c** Representative images of the RV cryosections stained with Masson’s trichrome (× 200). Collagen fibres are stained blue, nuclei are stained dark purple, and cytoplasm is stained red/pink, the scale bar is 100 µm. **d** Quantitative analysis of the RV cardiomyocyte cross-sectional area that was WGA-stained (µm^2^). **e** Quantitative analysis of the average CVF from the Masson’s trichrome-stained section in the RV (%). *WGA* wheat germ agglutinin, *CVF* collagen volume fraction. N = 5 per group. The horizontal lines show the mean ± SEM. One-way ANOVA. ^*^p < 0.05, ^**^p < 0.01, ^***^p < 0.001

Figure 1a shows the representative results of PAP and RVP wave images from each group. Compared with the CTL group, the MCT group exhibited a higher mean PAP (51.75 ± 4.53 mmHg in the MCT group vs. 18.94 ± 2.13 mmHg in the CTL group, *p* < 0.05) (Fig. 1b), mean RVP (36.77 ± 1.90 mmHg in the MCT group vs. 11.72 ± 1.72 mmHg in the CTL group, *p* < 0.05) (Fig. 1c), NT-pro-BNP level (1.37 ± 0.36 ng/mL in the MCT group vs. 0.19 ± 0.05 ng/mL in the CTL group, *p* < 0.05) (Fig. 1d), and Fulton index (0.56 ± 0.03% in the MCT group vs. 0.27 ± 0.02% in the CTL group, *p* < 0.05) (Additional file 6: Table S1).However, treatment with DAPA resulted in marked attenuation of PAH-induced pathophysiological changes (all *p* < 0.05). Additional file 1: Fig. S1 shows the representative echocardiographic images of RV and tricuspid orifice blood reflux in the 4 groups. The characteristics of the echocardiographic parameters of each group are summarised in the Additional file 7: Table S2. Compared with the CTL group, the MCT group had higher RV length, RV width, and PAD values. However, it had a lower PAAT, TAPSE, and RVFAC values. Treatment with low- and high-dose DAPA resulted in the prevention of the remodelling of PA and RV in rats with MCT injection (all *p* < 0.05). There were no significant differences in terms of CO, LVEDV, LVESV, or LVEF among the 4 groups (all *p* > 0.05). Consistent with these results, the histopathology of the heart sections showed that the average myocardial interstitial CVF and RVCM cross-sectional areas were significantly increased in the MCT group. However, low- and high-dose DAPA administration ameliorated myocardial fibrosis and hypertrophy in the RV with PAH-induced RHF (Fig. 2; all *p* < 0.05). To investigate the RV capillary density, the expression of CD31 was determined by immunohistochemical staining. Our results showed that compared with that in the CTL group, CD31 expression was significantly decreased in the MCT group (*p* < 0.05) but was not altered in the RV in the low- and high-dose DAPA-treated MCT-treated rats (all *p* > 0.05) (Additional file 3: Fig. S3). As illustrated in Additional file 4: Fig. S4a**,** TUNEL staining was used to detect CM apoptosis in the RVs of rats. The results of TUNEL staining showed that the apoptosis of RVCMs was markedly higher in the MCT group than in the CTL group (*p* < 0.001). However, DAPA treatment significantly decreased the apoptotic rate of RVCMs in both the MCT + LD and MCT + HD groups (Additional file 4: Fig. S4b; all *p* < 0.001 vs. MCT group).

### Effect of DAPA on PA histology and proliferation

Additional file 2: Fig. S2a shows the typical H&E staining images of PA from each group. Compared with those in the CTL group, the medial wall thickness, and medial wall area of the PA were significantly elevated in the MCT group on the other hand, DAPA treatment resulted in attenuation of these pathological changes (Additional file 2: Fig. S2d, e; all *p* < 0.05). In the present study, immunofluorescence staining of proliferating cell nuclear antigen (PCNA) was performed to assess the proliferation of PA smooth muscle cells. As shown in Additional file 2: Fig. S2b–d, the percentage of PCNA positive cells in the PA was significantly increased by 24.4% in the MCT group when compared with that in the CTL group (*p* < 0.001). However, low- and high-dose DAPA administration reduced the percentage of PCNA-positive cells by 10.8% and 18.2%, respectively (Additional file 2: Fig. S2g; all *p* < 0.05 vs. MCT group).

### ***Changes in the expression of key Ca***^***2***+^***handling proteins in the RV***

**Fig. 3:**
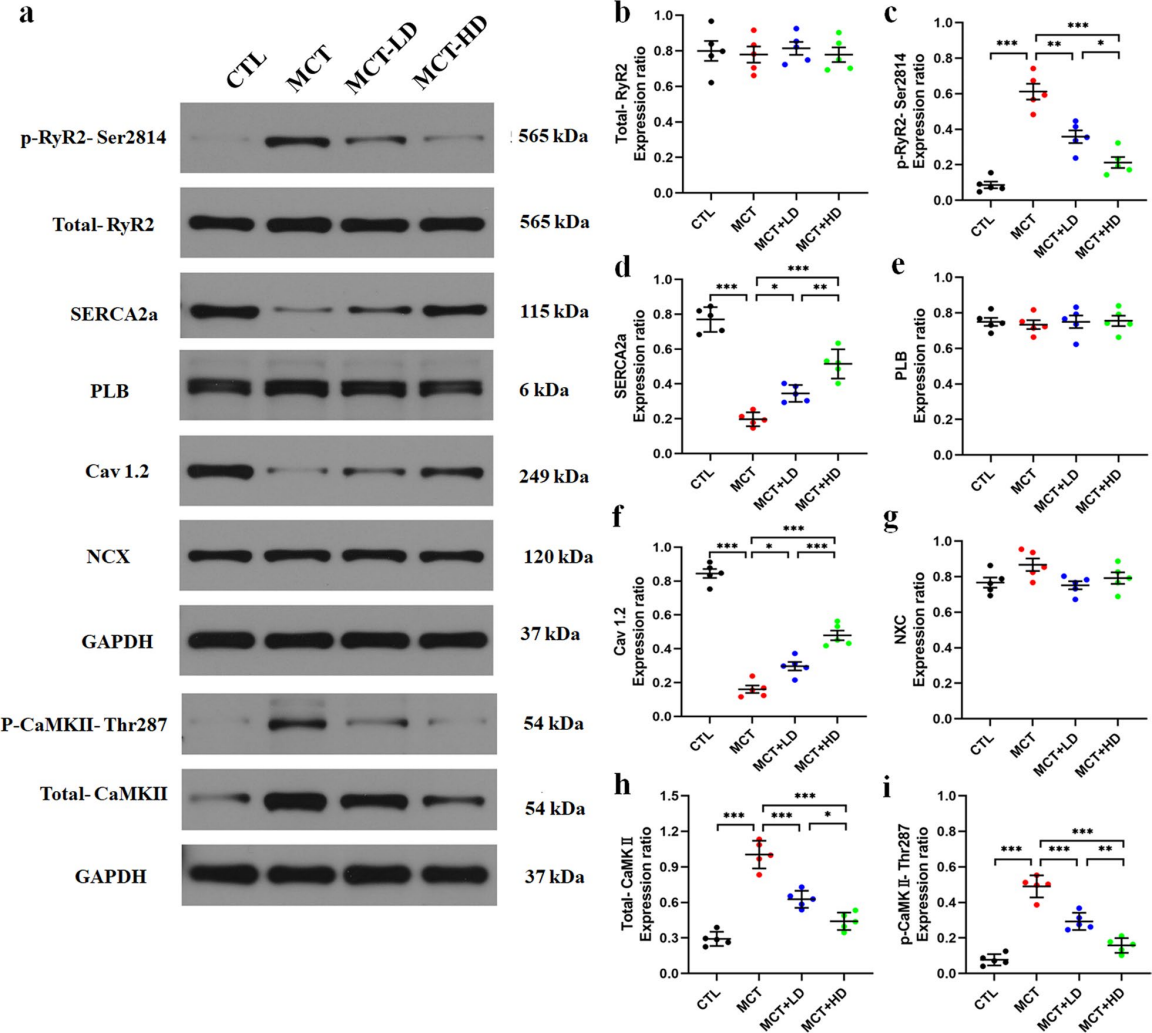
Western blot analysis. Total RyR2 (t-RyR2), phosphorylated RyR2 (p-RyR2-Ser2814), SERCA2a, total CaMKII (t-CaMKII), p-CaMKII (P-CaMKII-Thr287), phospholamban (PLB), Cav1.2, and Na^+^–Ca^2+^ exchanger protein (NCX) levels in the RV tissue from the 4 groups of rats were analysed and normalised to those of glyceraldehyde-3-phosphate dehydrogenase (GAPDH). N = 5 per group. The horizontal lines show the mean ± SEM. One-way ANOVA. ^*^p < 0.05, ^**^p < 0.01, ^***^p < 0.001

As shown in Fig. 3, there was a significant increase in the expression of p-RyR2, CaMKII, and p-CaMKII and marked decreases in the protein levels of SERCA2a and Cav1.2 in the MCT group compared with the CTL group (all *p* < 0.05). However, low-and high-DAPA treatment resulted in preservation of the protein levels of SERCA2a and Cav 1.2 and a reduction in the expression of p-RyR2, CaMKII, and p-CaMKII. There were no significant differences in the expression of RyR2, PLB, and NCX among the 4 groups (all *p* > 0.05).

### ***Effects of DAPA on intercellular Ca***^***2***+^***handling in RVCMs from MCT-treated rat hearts***

**Fig. 4:**
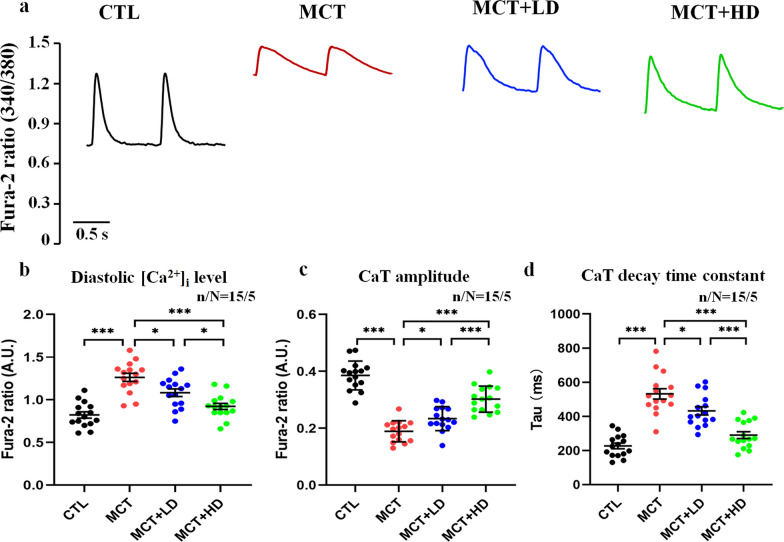
Ca^2+^ microfluorometry. **a** Recordings of CaTs from RVCMs in each group. **b** The diastolic [Ca^2+^]_i_ level, **c** CaT amplitude, and **d** CaT decay time constant (Tau) of a single RVCM were recorded and quantitatively analysed for RVCMs from the 4 groups of rats. Each point represents the result from a single RVCM. *RVCM* right ventricular cardiomyocyte, *CaT* Ca^2+^ transient. n/N = 15/5 = cells/rats per group. The horizontal lines show the mean ± SEM. One-way ANOVA. ^*^p < 0.05, ^**^p < 0.01, ^***^p < 0.001

Figure 4a shows the recordings of CaTs from RVCMs in each group. Compared with those in the CTL group, the diastolic [Ca^2+^]_i_ level (Fig. 4b) and CaT decay time constant (Fig. 4d) in the MCT group were markedly augmented. However, the CaT amplitude was significantly decreased in the MCT group (Fig. 4c) (all *p* < 0.05). In contrast, low- and high-dose DAPA administration resulted in significant reductions in the diastolic [Ca^2+^]_i_ level and CaT decay time constant. However, low- and high-dose DAPA administration also led to an increase in the CaT amplitude in RVCMs from MCT-treated rat hearts (all *p* < 0.05).

**Fig. 5:**
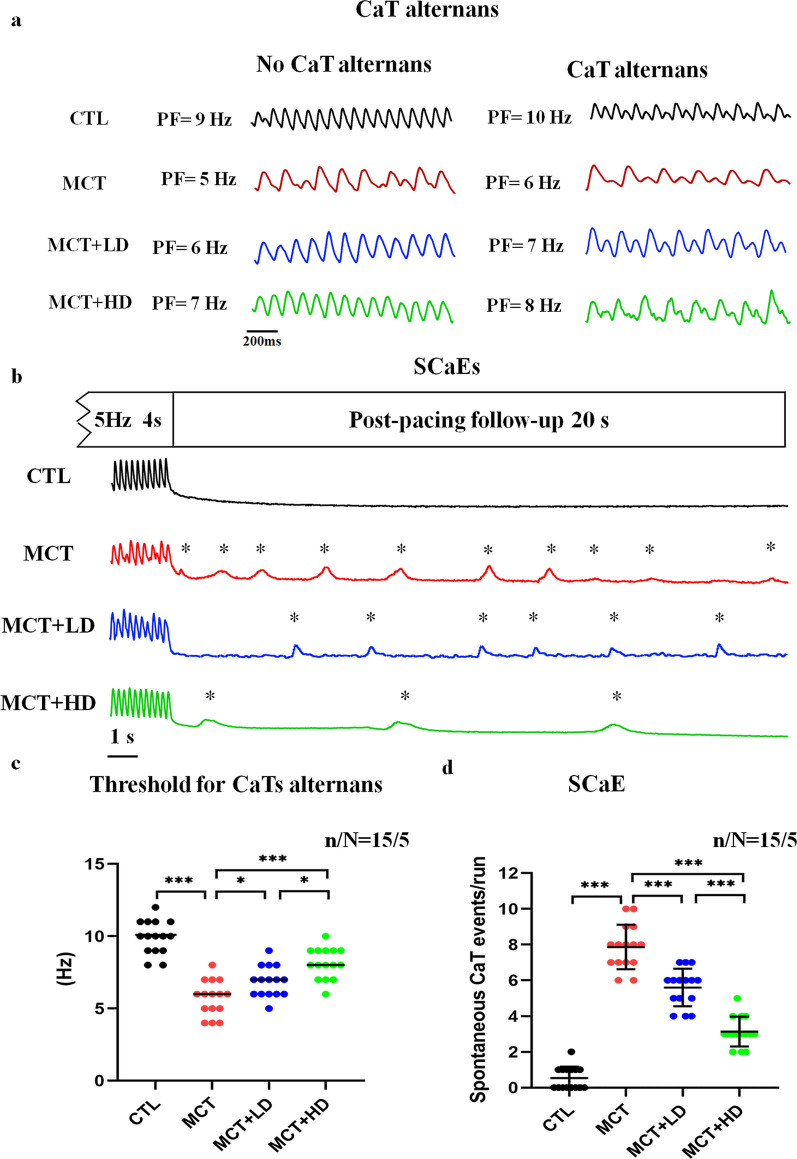
Assessment of CaT alternans and SCaEs. **a** Representative image of signals at progressively greater frequencies with and without the record of the alternans. The results from one cell in each group are shown in a column. CaT alternans is defined as 4 or more consecutive CaTs with more than a 5% change in amplitude. **b** Recording of SCaEs by 4 s at 5-Hz pacing and post-pacing under each condition from a single RVCM for the 4 groups of rats. **c** Threshold for cellular CaT alternans. **d** Calculation of the number of SCaEs per cell. Each point represents the result from a single RVCM. *RVCM* right ventricular cardiomyocyte, *CaT* Ca^2+^ transient, *SCaEs* spontaneous calcium events, *PF* pacing frequency. n/N = 15/5 = cells/rats per group. One-way ANOVA or the nonparametric Wilcoxon signed-rank test. ^*^p < 0.05, ^**^p < 0.01, ^***^p < 0.001

Cellular CaT alternans was induced at progressively higher frequencies in RVCMs from each group (Fig. 5a). The frequency threshold for CaT alternans in each RVCM from each group is shown in Fig. 5c. The CaT alternans frequency threshold was significantly reduced by 44.4% in RVCMs from the MCT group compared with those from the CTL group. In contrast, the CaT alternans frequency threshold was markedly increased in the DAPA-treated groups (by 40.0% in MCT + LD and by 60.0% in MCT + HD all *p* < 0.05).

We also recorded the SCaEs in RVCMs from each group (Fig. 5b). Compared with CTL RVCMs, more SCaEs were observed in RVCMs from MCT-treated hearts after the cessation of field stimulation. This finding is consistent with the SCaS results. Low-and high-dose DAPA administration indeed significantly prevented the SCaEs in RVCMs from MCT-treated rat hearts (Fig. 5d all *p* < 0.001).

**Fig. 6:**
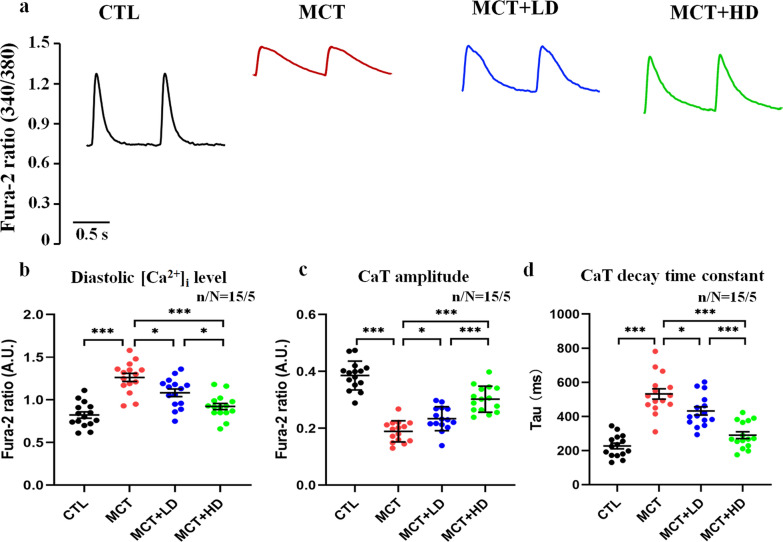
Assessment of [Ca^2+^]_SR_ by measurement of caffeine-induced CaTs. **a** Representative recording of caffeine-induced CaTs from a single RVCM for the 4 groups (caffeine = 10 mmol/L), which was used to estimate the total [Ca^2+^]_SR_ content. **b** CaT amplitude. **c** CaT decay time constant. Each point represents the result from a single RVCM. *RVCM* right ventricular cardiomyocyte, *CaT* Ca^2+^ transient, *[Ca*^*2*+^*]*_*SR*_ sarcoplasmic reticulum Ca^2+^ content. n/N = 15/5 = cells/rats per group. The horizontal lines show the mean ± SEM. One-way ANOVA or the nonparametric Wilcoxon signed-rank test. ^*^p < 0.05, ^**^p < 0.01, ^***^p < 0.001

Changes in [Ca^2+^]_SR_ were assessed by measuring caffeine-induced CaT. Figure 6a shows representative recordings of caffeine-induced CaT in RVCMs from each group. The [Ca^2+^]_SR_ was significantly decreased in RVCMs from the MCT group compared with those from the CTL group (*p* < 0.001). However, low- and high-dose DAPA administration resulted in the preservation of the [Ca^2+^]_SR_ in RVCMs from hearts that were MCT-treated (Fig. 6b all *p* < 0.01). There were no significant differences in the decay kinetics of caffeine-induced CaT among the 4 groups (Fig. 6c all *p* > 0.05).

**Fig. 7:**
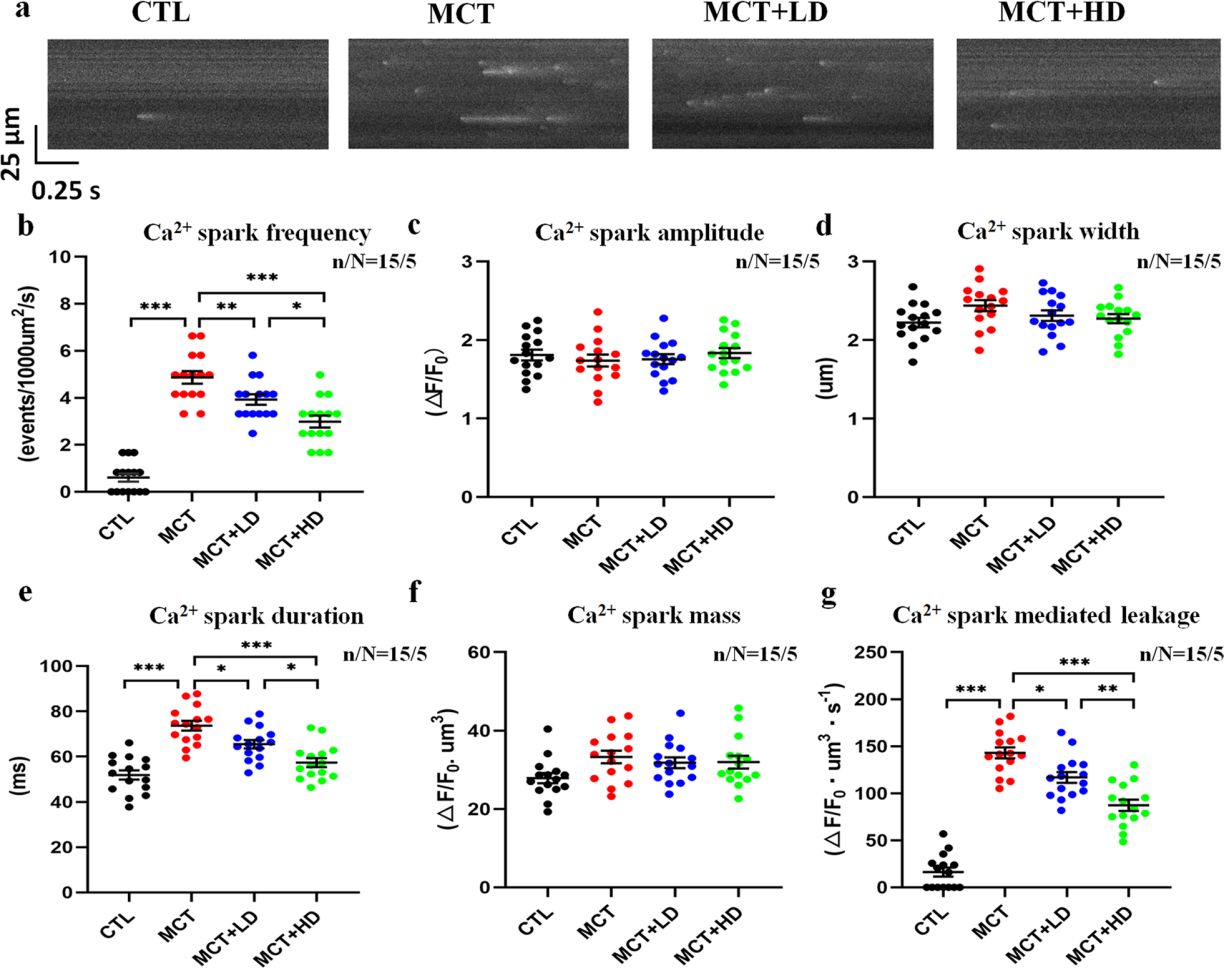
Assessment of the SCaS properties. **a** Representative image of the SCaS. **b** Ca^2+^ spark frequency. **c** Ca^2+^ spark amplitude. **d** Ca^2+^ spark width. **e** Ca^2+^ spark duration. **f** Ca^2+^ spark mass. **g** Ca^2+^ spark-mediated leakage. All parameters were recorded and quantitatively analysed from the RVCMs of the 4 groups of rats. Each point represents the result from a single RVCM. *RVCM* right ventricular cardiomyocyte, *SCaS* spontaneous Ca^2+^ sparks. n/N = 15/5 = cells/rats per group. The horizontal lines show the mean ± SEM. One-way ANOVA. ^*^p < 0.05, ^**^p < 0.001, ^***^p < 0.001

Since the behaviour of spontaneous Ca^2+^ release has been implicated in the changes in alternans [39], we quantified cellular SCaEs using confocal microscopy. Figure 7a shows the representative original recordings of SCaS at rest from each group. Compared with those in RVCMs from the CTL group, the SCaS frequency, SCaS duration, and SCaS-mediated leakage in RVCMs from the MCT group were markedly increased (all *p* < 0.05). However, low- and high-dose DAPA administration resulted in significant reductions in SCaS frequency, SCaS duration, and SCaS-mediated leakage in RVCMs from MCT-treated rat hearts (Fig. 7b, e, and g all *p* < 0.05). There was no statistically significant difference in the SCaS width among the 4 groups (Fig. 7c, d, and f all *p* > 0.05).

### *Effect of DAPA on RV electrophysiological characteristics *in vitro

**Fig. 8:**
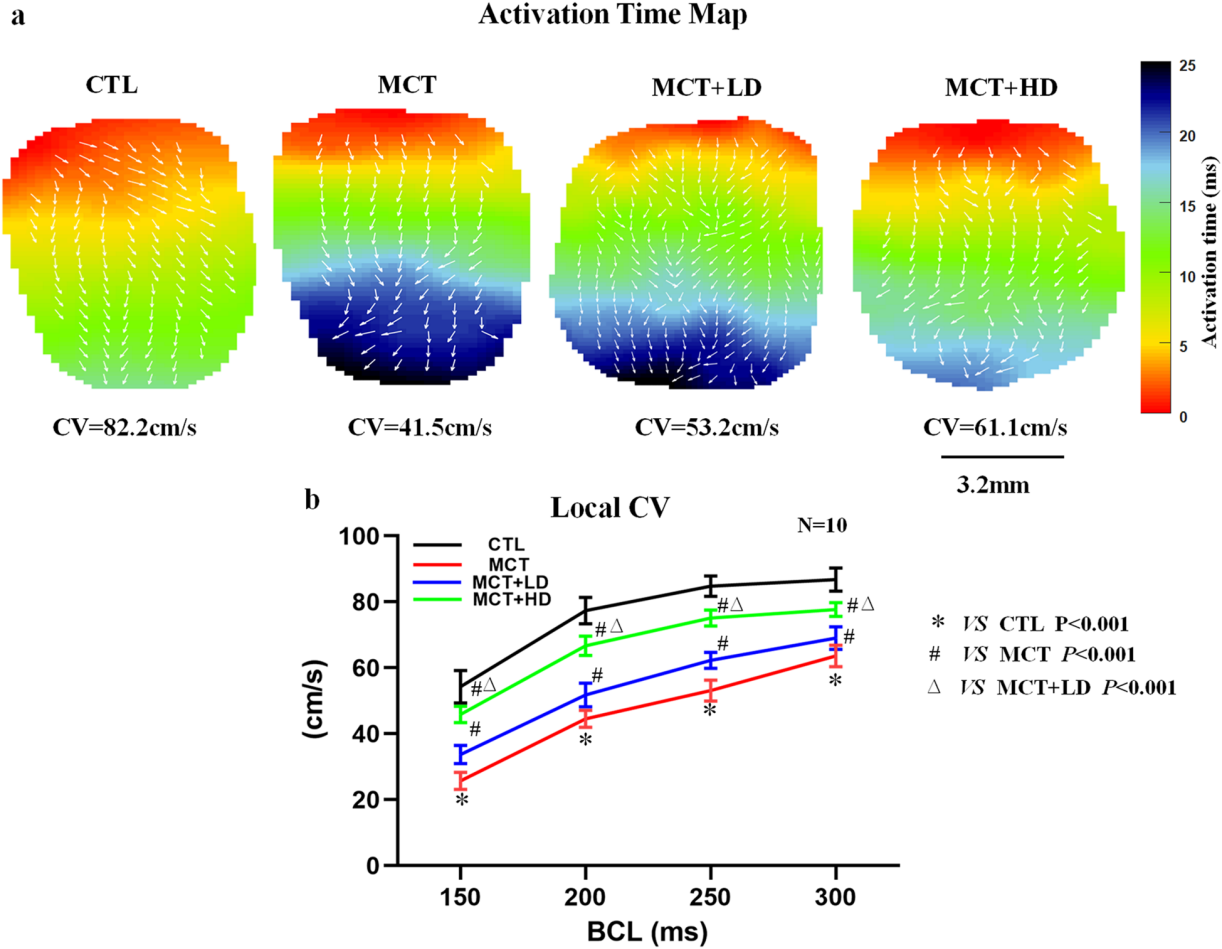
Optical mapping study of the RV. **a** Representative results of the activation time map at BCL 5 Hz (200 ms) in the RV free wall for the 4 groups. The arrow indicates the conduction direction. Right bar: colour-coded activation times across a representative RV surface. **b** Local CV in the four groups (cm/s). *BCL* basic cycle length, *CV* conduction velocity. n = 10 per group. The horizontal lines show the mean ± SEM. One-way ANOVA

Figure 8a shows the typical RV activation time maps recorded in isolated hearts from the 4 groups. The CV was significantly decreased at all coupling intervals below 200 ms in MCT-treated RVs compared with CTLs, but this effect of MCT was reversed by DAPA treatment in the MCT + LD and MCT + HD groups (Fig. 8b; all *p* < 0.001). To further explore the mechanisms by which DAPA affects CV alterations in hearts with PAH-induced RHF, as shown in Additional file 5: Fig. S5a, the degradation and disorganization of Cx43 were observed in the MCT group. However, compared with the value in the MCT group, DAPA treatment led to markedly increases in the PEA of Cx43 by 43% in the MCT + LD group and 59% in the MCT + HD group (Additional file 5: Fig. S5b; all *p* < 0.05).

**Fig. 9:**
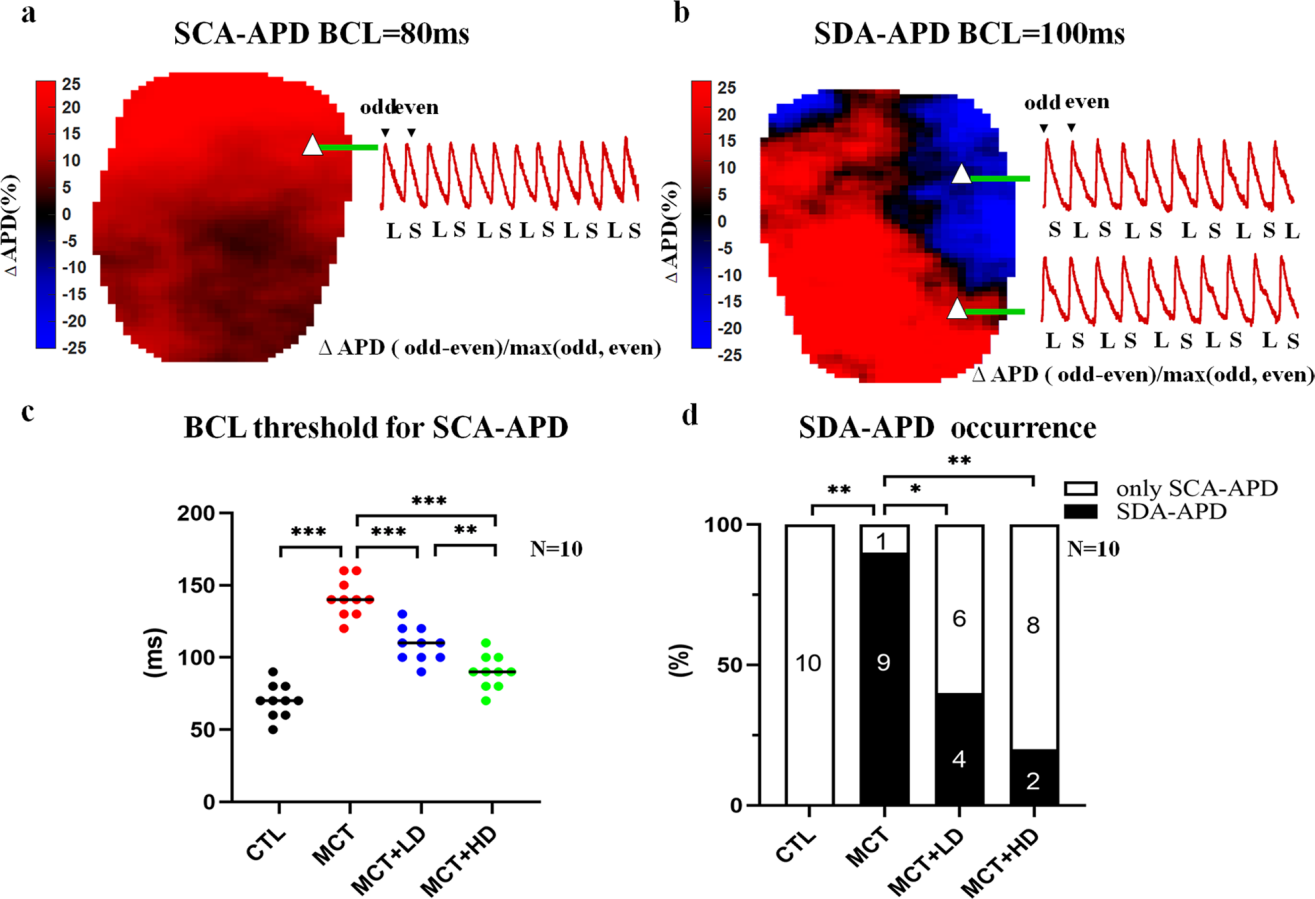
Assessment of APD alternans. **a** Representative recording of SCA-APD from a CTL heart. ΔAPD map and an AP recording from a single pixel at 80 ms BCL (position shown with a triangle on the map). **b** Example of the SDA-APD from an MCT heart. ΔAPD map and AP recordings from two pixels at 100 ms BCL. **c** The BCL threshold for SCA-APD alternans in each group. Each point represents the result from one heart. The horizontal lines show the median values for BCL that induce the SCA-APD alternans. **d** Inducibility of SDA-APD in each group. *SCA-APD* spatially concordant alternans action potential duration, *SDA-APD* spatially discordant alternans action potential duration, *AP* action potential, *BCL* basic cycle length. n = 10 per group. One-way ANOVA or *χ*^2^ tests. ^*^p < 0.05, ^**^p < 0.01, ^***^p < 0.001

We evaluated the effects of PAH-induced RHF on RVCM alternans in situ via optical mapping of APD alternans. Figure 9a shows the APD alternans thresholds in a CTL RV. Recordings from a single pixel showed that alternans in the whole CTL RV were in-phase and therefore concordant. Consistent with the single-RVCM results, the median BCL thresholds for APD alternans were significantly increased in the MCT-treated group compared with the CTL RVs (70 ms in the CTL group vs. 140 ms in the MCT group, Fig. 9c; *p* < 0.001). Moreover, Fig. 9b shows the APD alternans in two regions of the same RV from an MCT-treated heart. Out-of-phase alternans in the two regions were observed. This finding indicated that SDA-APD was elicited in the RV. In this study, the SCA-APD was induced in both the CTL and MCT groups. The SDA-APD occurred in 90% of RVs in the MCT groups, while it occurred in 0% of the CTL RVs (Fig. 9d; *p* < 0.05).

DAPA treatment led to significant decreases in the BCL thresholds for APD alternans in MCT-treated RVs from a median of 140 ms in the MCT group to a median of 110 ms in the MCT + LD group and 90 ms in the MCT + HD group (Fig. 9c all *p* < 0.05). Additionally, compared with the value in the MCT group, DAPA treatment marked reduced the inducibility of SDA-APD by 50% in the MCT + LD group and 70% in the MCT + HD group (Fig. 9d; all *p* < 0.05).

**Fig. 10:**
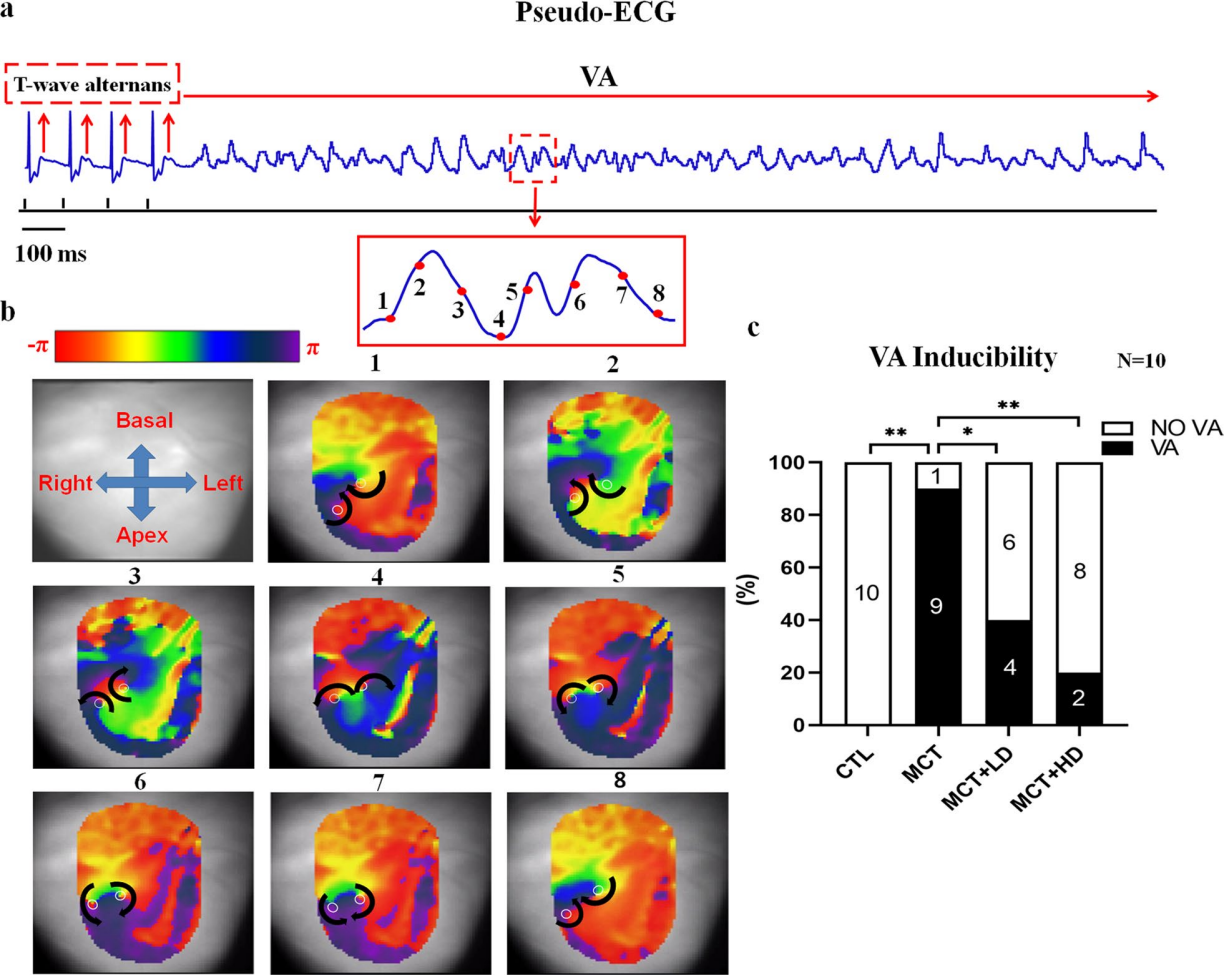
Detection and analysis of VA in the four groups of rats. **a** Pseudo-ECG before and during VA initiated by pacing at 100-ms BCL. T-wave alternans (shown with arrows) was found prior to VA initiation. **b** The panels show eight snapshots of the phase maps, which correspond to the time points indicated in red points of the amplified pseudo-ECG episode. The positions of phase singularities (PSs) are indicated by the white circle. The formation of the rotor, a hallmark of re-entrant VA, was observed in MCT-treated RVs. **c** Inducibility of VA in each group. *VA* ventricular arrhythmia, *BCL* basic cycle length, n = 10 per group. *χ*^2^ tests. ^*^p < 0.05, ^**^p < 0.01

Figure 10a shows a pseudo-ECG of VA induction by dynamic pacing in optically mapped MCT hearts. Phase mapping analysis of action potentials at the onset of VA was performed to reveal evidence of the re-entrant activity that maintained VA. The stable rotor activity initiating VA is illustrated in Fig. 10b, in which the phase singularities of the rotor at eight snapshots of phase maps covering a rotational cycle are shown. In our optical mapping study, VA was induced in 90% (9/10) of the MCT-treated RVs compared with 0% (0/10) of the CTL RVs. In contrast, the inducibility of VA in MCT-treated hearts was significantly suppressed to 40% (4/10) following DAPA treatment in the MCT + LD group and to 20% (2/10) in the MCT + HD group (Fig. 10c; all *p* < 0.05).

## Discussion

### Main finding

In this study, we examined the effects of DAPA treatment on cellular Ca^2+^ handling and ventricular arrhythmogenesis in rats with PAH-induced RHF. Our results indicate that (1) treatment with DAPA reduces PAP and RVP, attenuates RV structural and functional remodelling, inhibits the apoptosis of RVCMs, prevents PA smooth muscle cell proliferation, and preserves the expression of Cx43 in rats with MCT injection (2) treatment with DAPA restores Ca^2+^ handling in RVCMs isolated from hearts with MCT stimulation by increasing the CaT amplitude, decreasing the diastolic [Ca^2+^]_i_ level and CaT decay time constant, preserving the [Ca^2+^]_SR_, preventing SCaS and SCaEs, and augmenting the threshold for Ca^2+^ alternans (3) treatment with DAPA leads to marked increases in the thresholds of CV and the BCL for APD alternans but reduces the vulnerability to SCA-APD and VA in MCT-treated RVs and (4) DAPA administration results in significant preservation of SERCA2a and Cav 1.2 and reductions in the p-RyR2, PLB, CaMKII, and p-CaMKII protein levels in MCT-treated RVs.

### Effects of DAPA treatment on PAH-induced RHF

PAH-induced RHF in rats has been widely used for studying the efficacy of therapeutic agents for RHF treatment [40, 41]. Consistent with the findings of previous studies [6, 36], we also observed that MCT administration led to PA remodelling and RHF in rat hearts. This was manifested by the increased PAP, PA wall thickness, PAD, RV length, RV width, RVP, and serum NT-pro-BNP level and decreased PAAT, TAPSE, and RVFAC. However, DAPA treatment attenuated these changes in echocardiographic and anatomic parameters and the serum NT-pro-BNP level, suggesting that DAPA could ameliorate PA remodelling and RV dysfunction in MCT-treated rat hearts. In this study, DAPA treatment reduced the apoptosis in RVCMs from MCT-treated hearts. Previous studies have confirmed that apoptosis plays a key role in RV remodelling and eventually leads to RHF by increasing the loss of terminally differentiated cardiomyocytes [42–44]. In rats with MCT-induced PAH, Zou et al. [45] found that nicorandil prevented RV remodelling partly by inhibiting RVCM apoptosis. Similarly, Campian et al. [46] reported that valsartan reduced RVCM apoptosis and thereby delayed the deterioration of RV function in rats with MCT-induced RHF. Thus, we speculated that the anti-apoptotic role contributed to the improvement of RV function elicited by DAPA in this study.

It is well known that abnormal intercellular Ca^2+^ handling can result in cardiac remodelling and dysfunction [47]. Normalisation of intercellular Ca^2+^ handling has been shown to prevent PAH-induced RHF. Xie et al. [48] reported that sildenafil prevented Ca^2+^ handling dysfunction and hence reversed RV contractile failure in a model of PAH-induced RHF. Similarly, Fowler et al. [21] found that metoprolol treatment resulted in attenuation of PAH-induced RV failure by improving Ca^2+^ handling in RVCMs. In the present study, DAPA treatment led to a reduction in [Ca^2+^]_i_ overload, preservation of [Ca^2+^]_SR_, and a reduction in diastolic Ca^2+^ leakage in RVCMs from rats that were subjected to MCT stimulation. Therefore, we presume that restoration of Ca^2+^ homeostasis may have been an important mechanism for the prevention of PAH-induced RHF by DAPA treatment in our study.

Furthermore, in a rat model of MCT-induced PAH, Chowdhury et al. [49] demonstrated that administration of empagliflozin, a SGLT2i, resulted in abrogation of PA remodelling by reducing the rates of proliferation in the PA vascular wall. In the present study, we found that DAPA decreased the percentage of PCNA-positive cells in PAs from MCT-treated hearts, indicating a reduction in the proliferation of PA smooth muscle cells. Therefore, it is reasonable to suggest that modification of proliferation rates in PAs may have been involved in the attenuation of PA remodelling by DAPA treatment in this study.

### ***DAPA improved Ca***^***2***+^***handling in RVCMs isolated from MCT-treated hearts***

Previous studies have shown that RVCMs from rats with PAH-induced RHF have aberrant [Ca^2+^]_i_ homeostasis [6, 21, 50]. In the current study, there was also evidence of impaired Ca^2+^ handling in RVCMs from MCT-treated rat hearts, including increased diastolic [Ca^2+^]_i_ levels, decreased CaT amplitude and [Ca^2+^]_SR_, and a prolonged CaT decay time constant, These changes were all prevented by DAPA treatment. The SERCA2a pump is a key protein in intercellular Ca^2+^ handling. It can transport 92% of cytosolic Ca^2+^ back into the SR during diastole in rat CMs [51]. Dysfunction of the SERCA2a pump can lead to a rise in the diastolic [Ca^2+^]_i_ level, prolongation of CaTs, and depletion of [Ca^2+^]_SR_. On the other hand, the depletion of [Ca^2+^]_SR_ may subsequently induce a decrease in Ca^2+^ release during systole [51–53]_._

Meanwhile, downregulation of Cav 1.2 expression may lead to decreased L-type Ca^2+^ current and ultimately result in a reduction in systolic Ca^2+^ release [54]. Thus, in this study, the preservation of the protein levels of SERCA2a and Cav 1.2 may have been associated with DAPA treatment-associated restoration of Ca^2+^ handling in RVCMs from MCT-treated hearts.

Furthermore, consistent with previous studies [21, 50], we also observed an increase in SCaS frequency and SCaS-mediated Ca^2+^ leakage in RVCMs from MCT-treated hearts, which were prevented by DAPA treatment. Increased SCaS-mediated Ca^2+^ leakage can exacerbate [Ca^2+^]_i_ overload and ultimately result in disrupted Ca^2+^ homeostasis in CMs [47]. RyR2 is the key Ca^2+^ release channel located in the SR membrane. It plays a pivotal role in intracellular excitation–contraction coupling. It has been proposed that enhanced RyR2 activity can promote SCaS by increasing the opening rate of RyR2 during the resting period [26]. The RyR2 activity is affected by various factors and mechanisms, including changes in [Ca^2+^]_SR_ and phosphorylation modification [55]. CaMKII is a serine/threonine-protein kinase that participates in the regulation of myocardial Ca^2+^ homeostasis by interacting with many Ca^2+^ handling proteins. In murine and human failing CMs, Mustroph et al. [13] found that acute treatment with SGLT2i for 24 h markedly reduced CaMKII activity and CaMKII-dependent RyR2 phosphorylation at the site of Ser2814, which was also observed in the present study. CaMKII can be activated by elevated cytoplasmic Ca^2+^ concentrations, which facilitates calmodulin binding and autophosphorylation of CaMKII at the Thr286 site [56, 57]. In our study, the reduced diastolic [Ca^2+^]_i_ level in RVCMs caused by DAPA treatment could explain the downregulated expression of Thr286-p-CaMKII in the MCT + LD and MCT + HD groups. Additionally, there is strong evidence that RyR2 hyperphosphorylation by CaMKII at the site Ser2814 can result in increased RyR2 activation even under a low-[Ca^2+^]_SR_ condition. In contrast, inhibition of CaMKII reduces RyR2 activity and prevents consequent diastolic SCaS in animal CMs [58]. Thus, downregulation of CaMKII -dependent RyR2 phosphorylation may be an important mechanism of DAPA that leads to the restoration of [Ca^2+^]_i_ homeostasis in RVCMs from MCT-treated hearts.

Previous studies have evaluated the effects of SGLT2i on the cytosolic Ca^2+^ concentration in ventricular CMs. Mustroph et al. [13] reported that 24 h of exposure to empagliflozin significantly reduced diastolic [Ca^2+^]_i_ level in normal murine myocytes. Similarly, Baartscheer et al. [59] showed that empagliflozin treatment decreased diastolic [Ca^2+^]_i_ levels within 10 min. These studies demonstrated that empagliflozin decreases myocardial cytoplasmic Ca^2+^ levels via direct inhibition of the cardiac Na^+^/H^+^ exchanger (NHE). However, whether the impairment of myocardial NHE flux is involved in the reduction in diastolic [Ca^2+^]_i_ level caused by DAPA remains to be further investigated.

### DAPA decreased the vulnerability to repolarisation alternans and VA in hearts with PAH-induced RHF

Since the first report of ventricular repolarisation alternans in a rat model of PAH-induced RHF [6], increasing evidence has confirmed the occurrence of repolarisation alternans in experimental and clinical PAH-induced RHF [1, 60]. In this study, we demonstrated that DAPA treatment reduced the susceptibility of MCT-treated hearts to CaT/APD alternans. A wide range of studies have pointed to the crucial role of intracellular CaT alternans in the development of APD alternans [61, 62]. In addition, abnormal [Ca^2+^]_SR_ cycling has been proposed as a potential cause of the CaT/APD alternans in CMs [26]. A reduction in SERCA2a expression, which results in significant blunting of [Ca^2+^]_SR_ reuptake, is involved in the production of CaT/APD alternans in the normal heart [63, 64], whereas targeted SERCA2a gene transfer prevents the CaT/APD alternans and alternans-related arrhythmia in normal and failing hearts [6, 65].

Furthermore, previous studies have suggested that increased diastolic RyR2-mediated [Ca^2+^]_SR_ leakage is an important trigger of cellular CaT/APD alternans. In post-myocardial infarction dogs, Belevych et al. reported that elevated diastolic [Ca^2+^]_SR_ leakage due to redox modulation of RyR2 promoted the generation of Ca^2+^ alternans [66]. In atrial fibrillation-remodelled hearts, we recently confirmed that inhibition of increased RyR2-mediated [Ca^2+^]_SR_ leakage by dantrolene can suppress CaT/APD alternans and thereby decrease AF vulnerability [32]. In a computational modelling study, SCaS was found to promote CaT alternans via a spark-induced spark mechanism in both physiological and pathophysiological conditions [67]. Thus, the preserved SERCA2a expression and decreased diastolic [Ca^2+^]_SR_ leakage observed in this study might have contributed to the DAPA-mediated decrease in the vulnerability of MCT-treated hearts to CaT/APD alternans.

Repolarisation alternans, a well-known predictor of VA in patients with heart failure, can be either SCA or SDA [32, 68, 69]. As the pacing rate is increased, cells in different regions alternate in phase with each other. This is known as SCA. Although SCA is not necessarily arrhythmogenic, it is perceived as a prerequisite for SDA. Furthermore, it can be elicited in normal cardiac tissue. As opposed to SCA, SDA is more malignant. Additionally, SDA may potentially cause unidirectional block and reentry by producing steep spatial repolarisation gradients over short distances [37]. Moreover, previous studies have strongly supported the potential role of SDA in the promotion of re-entrant VA in an animal model of PAH-induced RHF [6, 70]. In our study, SCA was induced in all hearts, but SDA occurred in 0% of CTL hearts vs. 80% of MCT-treated hearts. In contrast, DAPA treatment markedly reduced the occurrence of SDA in MCT-treated hearts, which might have been the major mechanism by which DAPA reduced the vulnerability of hearts with PAH-induced RHF to VA.

### Other mechanisms by which DAPA suppressed VA in hearts with PAH-induced RHF

In the present study, we found that DAPA treatment attenuated the reduction in CV in MCT-treated RVs. Alterations in CV are well-known contributors to arrhythmogenesis. A severely slowed CV not only promotes the development of functional block lines that would initiate a re-entrant VA but also can shorten the wavelength to facilitate the stabilisation of the re-entrant circuit [70]. Previous studies have supported the hypothesis that disorganisation and degradation of Cx43 are important pathogenic pathways for CV abnormalities in MCT-treated hearts [71, 72]. Moreover, severe myocardial fibrosis can change the activation route and delay the CV of cardiac tissue [73], whereas therapeutic approaches targeting myocardial fibrosis suppress CV abnormalities and are beneficial in reducing the susceptibility to arrhythmia [74]. We observed that DAPA alleviated the remodelling of Cx43 and attenuated myocardial fibrosis in MCT-treated hearts. These were likely the main mechanisms of DAPA that preserved the CV and mediated the antiarrhythmic effect in this study (Additional file 8).

### Clinical implications

Since DAPA reduces the mortality and hospitalisation rates from cardiovascular events in people with left ventricular failure [75, 76], it is reasonable to conclude that DAPA may be a promising therapeutic drug for improving the outcomes of patients with right ventricular failure [77]. SCD is commonly observed in patients with PAH-induced RHF, and VA is thought to be one of the major causes [4, 78]. In the present study, we observed that DAPA reduced the vulnerability of the hearts of rats with PAH-induced RHF to VA by improving Ca^2+^ handling. This provides novel insight into the usefulness of DAPA as a potential antiarrhythmic drug for preventing VA in PAH-induced RHF. Our findings are consistent with those of recent clinical studies, which have demonstrated that DAPA reduces the risk of VA and prevents SCD in people with heart failure and reduced ejection fraction [79, 80].

### Study limitations

Although there were important discoveries in our study, there were also some limitations. First, we observed that DAPA alleviated the remodelling of Cx43 in hearts with PAH-induced RHF, but the mechanism remains unknown. A recent study demonstrated that DAPA could reverse the remodelling of Cx43 via an AMP-activated protein kinase (AMPK) pathway in post-infarcted rat hearts [81]. However, whether the activation of the AMPK-related pathway is associated with the DAPA-induced prevention of Cx43 remodelling in our study requires further investigation. Second, we observed that DAPA treatment led to a reduction in SCaS-mediated Ca^2+^ leakage, SCaEs, and [Ca^2+^]_i_ in RVCMs from MCT-treated hearts. Increased diastolic Ca^2+^ leakage and [Ca^2+^]_i_ overload can activate the NCX current and thereby promote trigger activity [26]. In our study, a patch-clamp study was not performed. This would have provided an avenue to evaluate the effects of DAPA on the NCX current and trigger activity. Third, the MCT in the MCT-induced PAH model is toxic, and studies have confirmed that MCT injection can lead to coronary arteriole thickening and biventricular myocarditis [82, 83]. In addition, one major shortcoming of the MCT-induced PAH rat model is that it cannot simulate the key features of severe PAH pathology, and most experimental treatments seem to ameliorate PAH and reverse the associated damage [16]. There is a need to explore the effects of DAPA on a model of Sugen 5416/hypoxia-induced PAH. Fourth, whether the amelioration of pulmonary arterial remodeling is contributed to the cardioprotective effect of DAPA requires further investigation. Future studies should be designed to assess the effects of DAPA treatment on cardiomyocytes (eg., H9C2) ex vivo or the PA banding-induced RHF model in vivo. Fifth, animal models do not reflect clinical benefits and cannot be used as a replacement for clinical trials, and the cardiac electrophysiological characteristics of the rats are different from those of humans. Thus, these experimental results cannot be directly applied to human hearts. Further work needs to be performed to investigate the effects of DAPA treatment on VA vulnerability in patients with PAH-induced RHF.

## Conclusion

In summary, the present study demonstrates the effects of DAPA administration on ventricular Ca^2+^ homeostasis and arrhythmogenesis in rats with PAH-induced RHF. These data suggest that DAPA reduces the vulnerability of hearts with PAH-induced RHF to VA by improving Ca^2+^ handling.

## Supplementary Information

**Additional file 1: Fig. S1**. Representative echocardiographic images from the four groups of rats after 35 days. **a**–**d** Twodimensional (2-D) parasternal short-axis view. **e**–**h** Representative images of the pulmonary artery outflow were obtained via Doppler. **i**–**l** Representative images of tricuspid orifice blood reflux. Blue means that there is blood reflux in the tricuspid orifice valve area. **m**–**p** Representative images of the M-mode traces of the LV (parasternal short-axis view). *RV* right ventricle, *LV* left ventricle, *IVS* interventricular septum.

**Additional file 2: Fig. S2**. Histological and morphological analysis of PA remodelling in the four groups of rats. **a** Representative images of PA remodelling detected by haematoxylin and eosin (H&E) staining in the lungs (x400); the scale bar is 100 μm. **e** Quantitative analyses of medial wall thickness of the PA in the four groups. **f** Quantitative analyses of medial wall area of the PA in the four groups. **b**, **c**, **d** Representative images of PCNA immunofluorescence staining of the PA. Green fluorescence represents PCNA-positive nuclei, and blue fluorescence represents the total nuclei of cells, which were observed in five randomly selected fields using a fluorescence microscope (x200); the scale bar is 100 μm. **g** Comparison of the number of the PCNA-positive cells relative to the total number of smooth muscle cells in the medial wall of the PA in the four groups. *PA* pulmonary artery, *PCNA* proliferating cell nuclear antigen. n = 5 per group. One-way ANOVA. *p < 0.05, **p < 0.001,***p < 0.001.

**Additional file 3: Fig. S3**. Detection of CD31 expression in the RVs from the four groups of rats by immunohistochemical staining. **a** Representative image of CD31 immunohistochemical staining (x400); the scale bar is 50 μm. CD31-positive areas were classified as those with any brown-stained individual endothelial cells or clusters of endothelial cells, which were considered capillaries, the capillary density was calculated as the average positive number of the vessels in one section. **b** Comparison of the RV capillary density among the four groups. *RV* right ventricle. n = 5 per group. One-way ANOVA. ***p < 0.001.

**Additional file 4: Fig. S4**. Detection of the apoptosis of RVCMs from the four groups of rats by TUNEL staining. **a** Representative TUNEL immunofluorescence staining images. Green fluorescence represents TUNEL-positive nuclei, and blue DAPI fluorescence represents the total nuclei of cells, which were observed in five randomly selected fields using a fluorescence microscope (x400); the scale bar is 50 μm. **b** Comparison of the number of apoptotic cells among the four groups. *RVCMs* right ventricular cardiomyocytes. n = 5 per group. One-way ANOVA. *p < 0.05, **p < 0.01, ***p < 0.001.

**Additional file 5: Fig. S5**. Detection of Cx43 expression in the RVs from the four groups of rats by immunohistochemical staining. **a** Expression of Cx43 in each group of rats (x400); the scale bar is 50 μm. The arrows mark the degradation and disorganisation of Cx43. **b** Comparison of the PEA of Cx43 between the four groups. N = 5 per group. *RV* right ventricle, *PEA* positive expression area. n = 5 per group. One-way ANOVA. **p <0.01, ***p <0.001.

**Additional file 6: Table S1**. Animal and organ characteristics in the four groups of rats.

**Additional file 7: Table S2**. Echocardiographic parameters of the four groups of rats.

**Additional file 8**: Supplemental methods.

## Data Availability

The datasets used and/or analyzed during the current study are available from the corresponding author on reasonable request.

## Abbreviations

SGLT-2i: Sodium-glucose co-transporter-2 inhibitor
DAPA: Dapagliflozin
PAH: Pulmonary arterial hypertension
RHF: Right heart failure
RV: Right ventricle
LV: Left ventricle
VA: Ventricular arrhythmia
VT: Ventricular tachycardia
VF: Ventricular fibrillation
[Ca^2+^]_i_: Intracellular Ca^2+^
[Ca^2+^]_SR_: Sarcoplasmic reticulum Ca^2+^ content
MCT: Monocrotaline
MCT + LD: Monocrotaline plus low dose dapagliflozin
MCT + HD: Monocrotaline plus high dose dapagliflozin
RVCM: Right ventricular cardiomyocyte
CM: Cardiomyocyte
SCD: Sudden cardiac death
LVESV: Left ventricular end-systolic volume
LVEDV: Left ventricular end-diastolic volume
CO: Cardiac output
RVFAC: Right ventricular fractional area change
PA: Pulmonary artery
PAD: Pulmonary arterial diameter
PAAT: Pulmonary arterial acceleration time
TAPSE: Tricuspid annular plane systolic excursion
LVEF: Left ventricular ejection fraction
PE: Pressure polyethylene
PAP: Pulmonary arterial pressure
RVP: Right ventricular pressure
NT-pro-BNP: N-terminal pro-B-type natriuretic peptide
H andE: Hematoxylin and eosin
WGA: Wheat germ agglutinin
APD: Action potential duration
BCL: Basic cycle length
PCL: Pacing cycle length
CL: Cycle length
CV: Conduction velocity
RyR2: Type 2 ryanodine receptor
SERCA2a: Sarcoplasmic/endoplasmic reticulum Ca^2+^ ATPase 2a
PLB: Phospholamban
CaMKII: Calcium/calmodulin-dependent protein kinaseII
CaT: Ca^2+^ transients
SR: Sarcoplasmic reticulum
SCaS: Spontaneous Ca^2+^ sparks
SCaE: Spontaneous Ca^2+^ event
