# Correction: Dapaglifozin reduces the vulnerability of rats with pulmonary arterial hypertension-induced right heart failure to ventricular arrhythmia by restoring calcium handling

**DOI:** 10.1186/s12933-022-01669-4

**Published:** 2022-10-31

**Authors:** Jinchun Wu, Tao Liu, Shaobo Shi, Zhixing Fan, Roddy Hiram, Feng Xiong, Bo Cui, Xiaoling Su, Rong Chang, Wei Zhang, Min Yan, Yanhong Tang, He Huang, Gang Wu, Congxin Huang

**Affiliations:** 1 Department of Cardiology, Renmin Hospital of Wuhan University, No. 238 Jiefang Road, Wuhan, 430060 People’s Republic of China; 2 Cardiovascular Research Institute, Wuhan University, 238 Jiefang Road, Wuhan, 430060 People’s Republic of China; 3 Hubei Key Laboratory of Cardiology, 238 Jiefang Road, Wuhan, 430060 People’s Republic of China; 4 Department of Cardiology, Qinghai Provincial People’s Hospital, No. 2 Gong He Road, Xining, 810007 People’s Republic of China; 5 Department of Medicine, Faculty of Medicine, Montreal Heart Institute (MHI), Universite de Montreal, Montreal, QC Canada; 6 Department of Cardiology, Shenzhen Longhua District Central Hospital, The Affiliated Central Hospital of Shenzhen Longhua District, Guangdong Medical University, No. 187 Guanlan Road, Longhua District, Shenzhen, 518109 China

## Correction: Cardiovascular Diabetology (2022) 21:197 https://doi.org/10.1186/s12933-022-01614-5

Following publication of the original article [1], the author noticed the error in Fig. 6. In the published article, Figs. 4 and 6 look same. The author has wrongly uploaded Fig. 6 in the manuscript package which has been processed by the typesetter. However, the text citations and caption of Fig. 6 seems to be correct. Now this has been corrected with this erratum. The corrected Fig. 6 has been given in this correction.

**Fig. 6:**
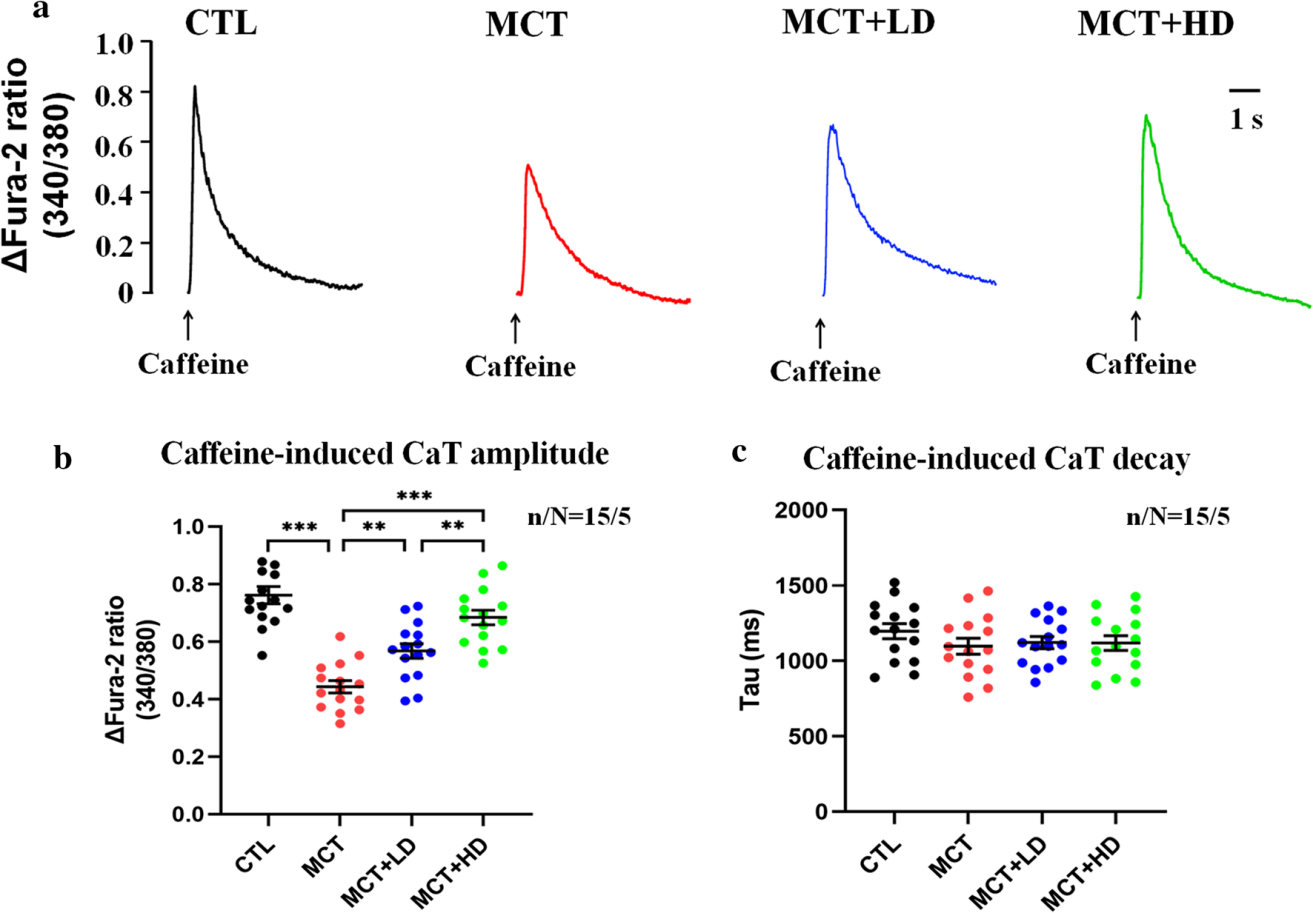
Assessment of [Ca^2+^]_SR_ by measurement of caffeine-induced CaTs. **a** Representative recording of caffeine-induced CaTs from a single RVCM for the 4 groups (caffeine = 10 mmol/L), which was used to estimate the total [Ca^2+^]_SR_ content. **b** CaT amplitude. **c** CaT decay time constant. Each point represents the result from a single RVCM. *RVCM* right ventricular cardiomyocyte, *CaT* Ca^2+^ transient, *[Ca*^*2*+^*]*_*SR*_ sarcoplasmic reticulum Ca^2+^ content. n/N = 15/5 = cells/rats per group. The horizontal lines show the mean ± SEM. One-way ANOVA or the nonparametric Wilcoxon signed-rank test. *p < 0.05, **p < 0.01, ***p < 0.001
