# Supplementary figures and images for: Dapagliflozin reduces the vulnerability of rats with pulmonary arterial hypertension-induced right heart failure to ventricular arrhythmia by restoring calcium handling
Source: Cardiovasc Diabetol. 2022 Sep 28;21:197. doi: 10.1186/s12933-022-01614-5 (PMC9516842; doi:10.1186/s12933-022-01614-5)

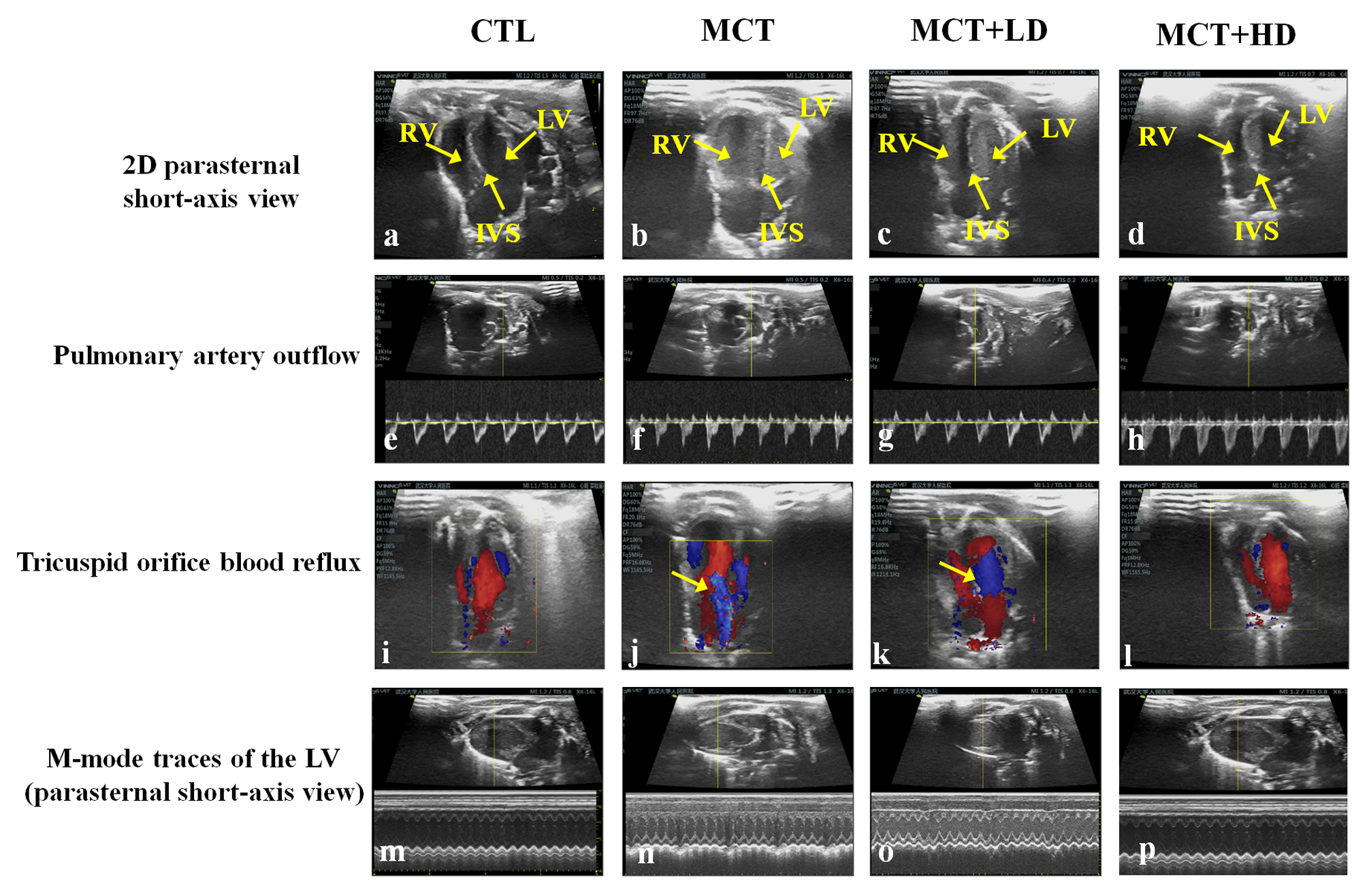

Supplement: Supplementary file 1 — Additional file 1: Fig. S1. Representative echocardiographic images from the four groups of rats after 35 days. a–d Twodimensional (2-D) parasternal short-axis view. e–h Representative images of the pulmonary artery outflow were obtained via Doppler. i–l Representative images of tricuspid orifice blood reflux. Blue means that there is blood reflux in the tricuspid orifice valve area. m–p Representative images of the M-mode traces of the LV (parasternal short-axis view). RV right ventricle, LV left ventricle, IVS interventricular septum. [file 12933_2022_1614_MOESM1_ESM.tif]

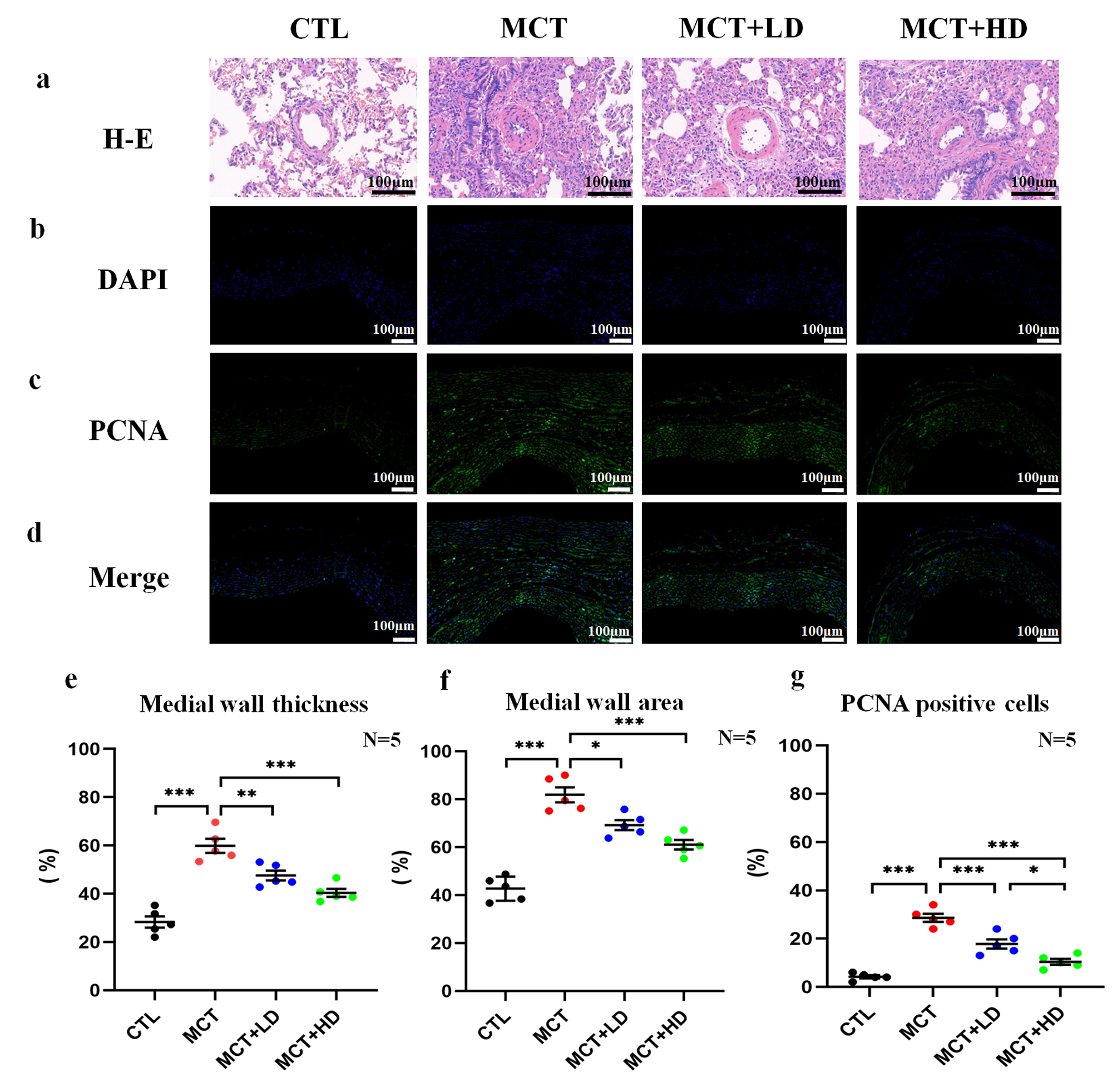

Supplement: Supplementary file 2 — Additional file 2: Fig. S2. Histological and morphological analysis of PA remodelling in the four groups of rats. a Representative images of PA remodelling detected by haematoxylin and eosin (H&E) staining in the lungs (x400); the scale bar is 100 μm. e Quantitative analyses of medial wall thickness of the PA in the four groups. f Quantitative analyses of medial wall area of the PA in the four groups. b, c, d Representative images of PCNA immunofluorescence staining of the PA. Green fluorescence represents PCNA-positive nuclei, and blue fluorescence represents the total nuclei of cells, which were observed in five randomly selected fields using a fluorescence microscope (x200); the scale bar is 100 μm. g Comparison of the number of the PCNA-positive cells relative to the total number of smooth muscle cells in the medial wall of the PA in the four groups. PA pulmonary artery, PCNA proliferating cell nuclear antigen. n = 5 per group. One-way ANOVA. *p < 0.05, **p < 0.001,***p < 0.001. [file 12933_2022_1614_MOESM2_ESM.tif]

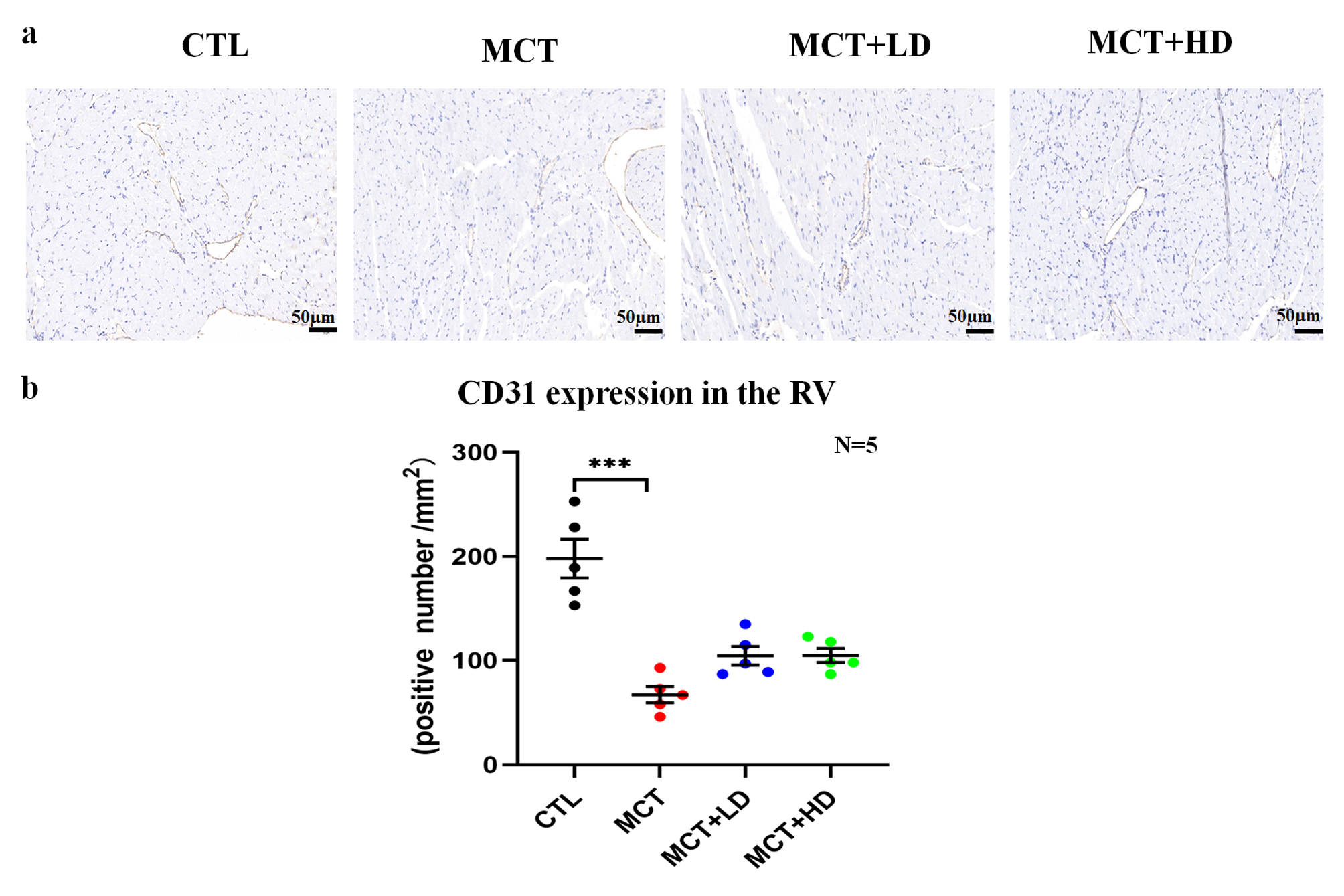

Supplement: Supplementary file 3 — Additional file 3: Fig. S3. Detection of CD31 expression in the RVs from the four groups of rats by immunohistochemical staining. a Representative image of CD31 immunohistochemical staining (x400); the scale bar is 50 μm. CD31-positive areas were classified as those with any brown-stained individual endothelial cells or clusters of endothelial cells, which were considered capillaries, the capillary density was calculated as the average positive number of the vessels in one section. b Comparison of the RV capillary density among the four groups. RV right ventricle. n = 5 per group. One-way ANOVA. ***p < 0.001. [file 12933_2022_1614_MOESM3_ESM.tif]

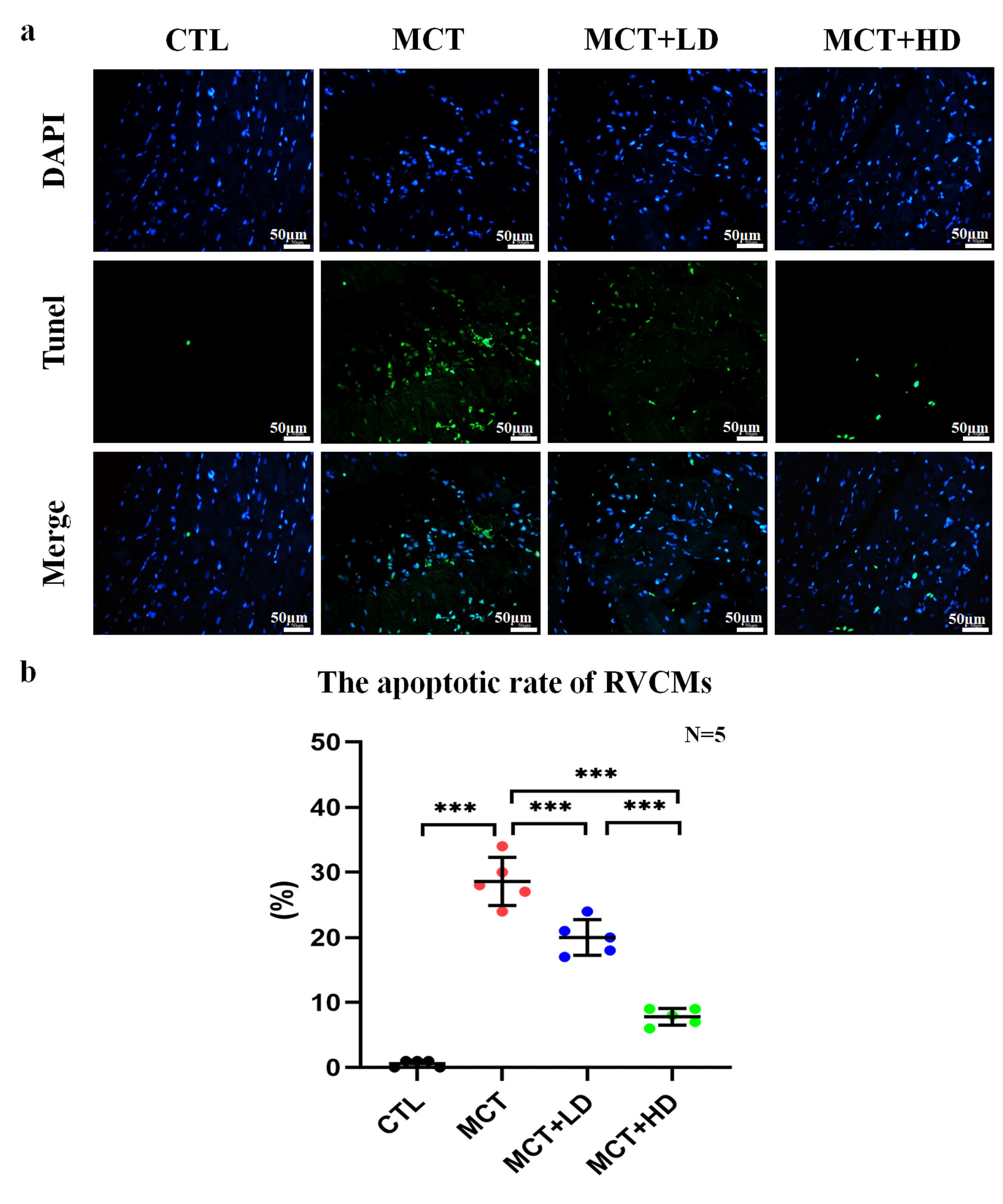

Supplement: Supplementary file 4 — Additional file 4: Fig. S4. Detection of the apoptosis of RVCMs from the four groups of rats by TUNEL staining. a Representative TUNEL immunofluorescence staining images. Green fluorescence represents TUNEL-positive nuclei, and blue DAPI fluorescence represents the total nuclei of cells, which were observed in five randomly selected fields using a fluorescence microscope (x400); the scale bar is 50 μm. b Comparison of the number of apoptotic cells among the four groups. RVCMs right ventricular cardiomyocytes. n = 5 per group. One-way ANOVA. *p < 0.05, **p < 0.01, ***p < 0.001. [file 12933_2022_1614_MOESM4_ESM.tif]

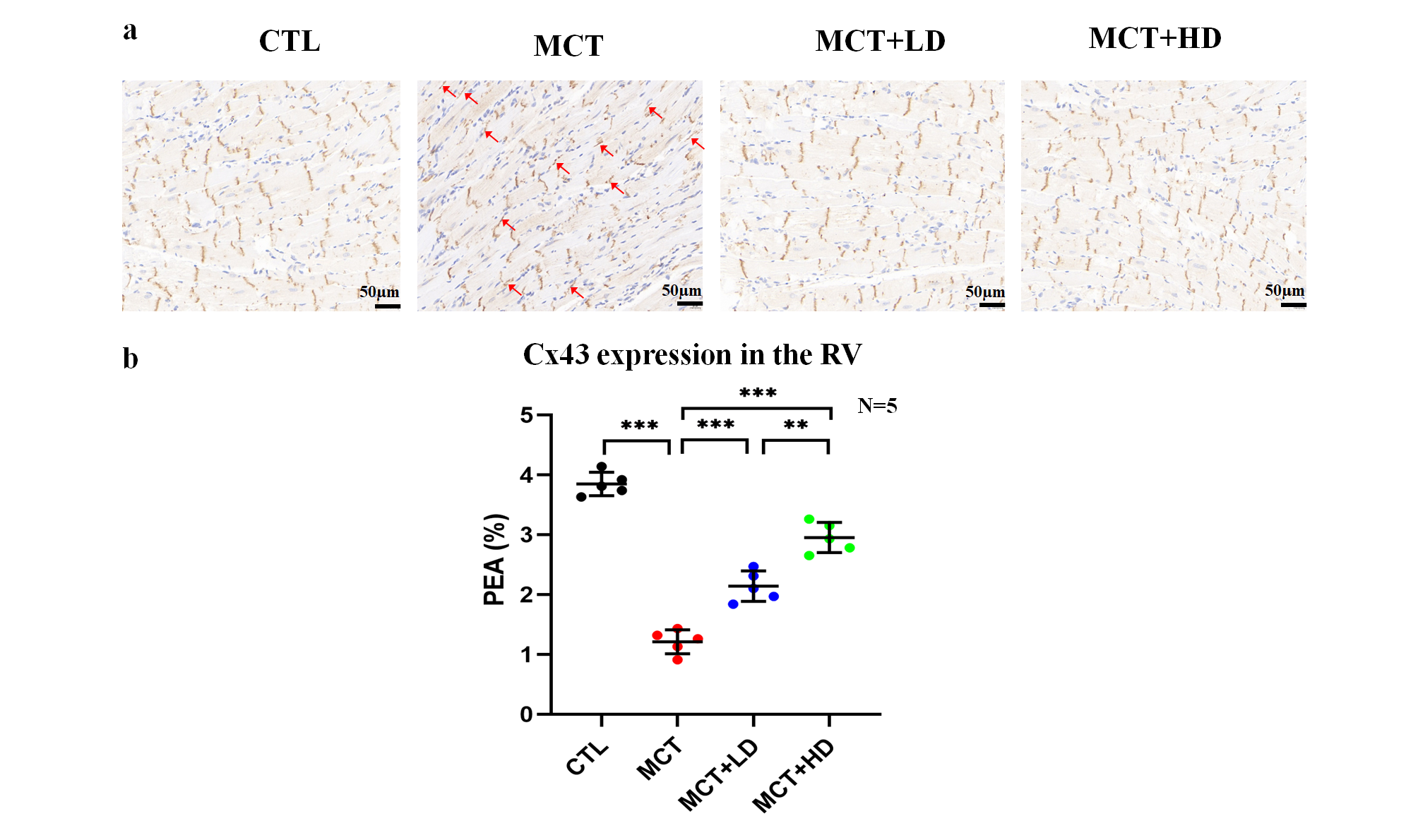

Supplement: Supplementary file 5 — Additional file 5: Fig. S5. Detection of Cx43 expression in the RVs from the four groups of rats by immunohistochemical staining. a Expression of Cx43 in each group of rats (x400); the scale bar is 50 μm. The arrows mark the degradation and disorganisation of Cx43. b Comparison of the PEA of Cx43 between the four groups. N = 5 per group. RV right ventricle, PEA positive expression area. n = 5 per group. One-way ANOVA. **p <0.01, ***p <0.001. [file 12933_2022_1614_MOESM5_ESM.tif]
