# Supplementary material for: Dapagliflozin reduces the vulnerability of rats with pulmonary arterial hypertension-induced right heart failure to ventricular arrhythmia by restoring calcium handling
Source: Cardiovasc Diabetol. 2022 Sep 28;21:197. doi: 10.1186/s12933-022-01614-5 (PMC9516842; doi:10.1186/s12933-022-01614-5)
Supplement: Supplementary file 6 — Additional file 6: Table S1. Animal and organ characteristics in the four groups of rats. [file 12933_2022_1614_MOESM6_ESM.doc]

**Additional file 6: Table S1.**

**The Animal and Organ Characteristics in the Four Groups of Rats**

|  | CTL | MCT | MCT+LD | MCT+HD |
| --- | --- | --- | --- | --- |
| BW on Baseline (g) | 301.25±9.57 | 293.63±8.38 | 295.63±9.84 | 303.25±8.22 |
| BW after 35 days (g) | 409.00±10.56 | 335.50±9.53^*^ | 347.50±10.25^*#^ | 356.63±10.99^*#^ |
| HW (mg) | 1387.50±41.19 | 1479.25±55.21^*^ | 1398.38±22.68^#^ | 1247.75±37.53^*#∆^ |
| HW/BW-35 days (g/g, %) | 0.34±0.01 | 0.44±0.02^*^ | 0.40±0.01^*#^ | 0.35±0.01^#∆^ |
| RVW (mg) | 297.00±19.30 | 531.13±22.60^*^ | 452.75±21.66^*#^ | 382.75±19.70^*#∆^ |
| LV+IVS (mg) | 1090.50±36.77 | 948.13±47.88^*^ | 945.63±15.65^*^ | 865.00±34.57^*#∆^ |
| Fulton Index (mg/mg, %) | 27.24±1.99 | 56.13±3.52^*^ | 47.90±2.63^*#^ | 44.32±3.08^*#^ |

*BW* body weight, *HW* heart weight, *RVW* right ventricular weight, *LV + IVS* left ventricular weight+ inter ventricular septum weight, *Fulton Index* RVW / (LV + IVS).

Data are presented as the *mean ± S.E.M. P* values were calculated using a one-way analysis of variance test and Tukey's multiple comparisons test used for multiple comparisons. N = 8 per group.

*^*^P*<0.05, indicates statistical significance compared with the CTL group.

*^#^P*<0.05, indicates statistical significance compared with the MCT group.

*^∆^P*<0.05, indicates statistical significance compared with the MCT+LD group.
