# Supplementary material for: Dapagliflozin reduces the vulnerability of rats with pulmonary arterial hypertension-induced right heart failure to ventricular arrhythmia by restoring calcium handling
Source: Cardiovasc Diabetol. 2022 Sep 28;21:197. doi: 10.1186/s12933-022-01614-5 (PMC9516842; doi:10.1186/s12933-022-01614-5)
Supplement: Supplementary file 7 — Additional file 7: Table S2. Echocardiographic parameters of the four groups of rats. [file 12933_2022_1614_MOESM7_ESM.doc]

**Additional file 7: Table. S2.**

**Echocardiographic Parameters in the Four Groups of Rats**

|  | CTL | MCT | MCT+LD | MCT+HD |
| --- | --- | --- | --- | --- |
| RV-width (mm) | 4.56±0.29 | 5.84±0.16^*^ | 5.55±0.18^*^ | 5.22±0.32^*#^ |
| RV-length (mm) | 9.50±0.60 | 11.41±0.16^*^ | 10.91+0.42 | 10.79±0.64 |
| PAD (mm) | 2.15±0.07 | 3.70±0.18^*^ | 3.56±0.28^*^ | 2.65±0.14^*#∆^ |
| PAAT (mm) | 36.25±3.07 | 21.4±2.41^*^ | 25.40±3.36^*^ | 28.20±1.92^*#∆^ |
| TAPSE (mm) | 2.96±0.16 | 2.21±0.12^*^ | 2.85±0.14^#^ | 2.82±0.10^#^ |
| RVFAC (%) | 44.93±2.86 | 27.02±1.60^*^ | 33.86±1.56^*#^ | 36.81±1.21^#^ |
| CO (ml/min) | 334.14±25.85 | 317.92±58.49 | 336.58±42.24 | 345.40±13.55 |
| LVEDV (ml) | 1.29±0.12 | 1.21±0.14 | 1.25±0.06 | 1.28±0.09 |
| LVESV (ml) | 0.24±0.04 | 0.24±0.08 | 0.23±0.09 | 0.19±0.02 |
| LVEF (%) | 81.30±3.08 | 83.42±3.07 | 81.49±6.34 | 90.31±4.77 |

*RV* right ventricle, *PAD* pulmonary arterial diameter, *PAAT* pulmonary artery acceleration time, *TAPSE* tricuspid annular plane systolic excursion, *RVFAC* right ventricular fractional area change, *CO* cardiac output, *LVEDV* left ventricular end-diastolic volume, *LVESV* left ventricular end-systolic volume, *LVEF* left ventricular ejection fractions.

Data are presented as the *mean ± S.E.M*. P values were calculated using a one-way analysis of the variance test. Tukey's multiple comparisons test was used for multiple comparisons. N = 5 per group.

*^*^P*<0.05, indicates statistical significance compared with the CTL group.

*^#^P*<0.05, indicates statistical significance compared with the MCT group.

*^∆^P*<0.05, indicates statistical significance compared with the MCT+LD group.
