# Supplementary material for: Dapagliflozin reduces the vulnerability of rats with pulmonary arterial hypertension-induced right heart failure to ventricular arrhythmia by restoring calcium handling
Source: Cardiovasc Diabetol. 2022 Sep 28;21:197. doi: 10.1186/s12933-022-01614-5 (PMC9516842; doi:10.1186/s12933-022-01614-5)
Supplement: Supplementary file 8 — Additional file 8: Supplemental methods. [file 12933_2022_1614_MOESM8_ESM.doc]

**Supplemental Methods**

**Histological and morphological analysis of the PA remodelling in the four groups of rats.**

Lung tissues were collected and fixed in 4% paraformaldehyde for 24 h, embedded in paraffin, and sectioned into 5 µm-thick sections. As previously described , the lung sections were stained with H&E staining and photographed (x400). Briefly, the percent medial wall thickness was calculated as (outside diameter - inside diameter) / (outside diameter) x100%, the percent medial wall area was calculated as (medial wall area) / (total vessel area) x100%, and PA remodelling was calculated by measuring the percent medial wall thickness and percent medial wall area of PA. For analyses of PA smooth muscle cell proliferation indices, the lung sections were stained with a proliferating cell nuclear antigen (PCNA) rabbit polyclonal antibody (GB11010, 1:400, Servicebio, China), and then the ratio of the PCNA-positive cells to the total cell number of cells in each PA was analysed as reported .

**Detection of the apoptosis of RVCMs from four groups of rats by the TUNEL staining.**

As previously described, RV myocardial apoptosis was evaluated via terminal deoxynucleotidyl transferase-mediated dUTP nick-end labelling (TUNEL) staining following the manufacturer’s instructions and as previously described. Briefly, TUNEL-positive cells were observed in five randomly selected fields using a fluorescence microscope (x400); the nucleus was blue, and positive apoptotic cells were green. The number of positive cells per high-power field was calculated and analysed in a blinded manner using Image-Pro Plus 6.0 software (Media Cybernetics, Inc). The apoptosis index (AI) was calculated as the percentage of apoptotic cell nuclei/total cell nuclei.

**References**

1. Jin H, Wang Y, Zhou L, Liu L, Zhang P, Deng W, Yuan Y. Melatonin attenuates hypoxic pulmonary hypertension by inhibiting the inflammation and the proliferation of pulmonary arterial smooth muscle cells. J Pineal Res. 2014; 57 (4): 442-50.

2. Jin H, Jiao Y, Guo L, Ma Y, Zhao R, Li X, Shen L, Zhou Z, Kim SC, Liu J. Astragaloside IV blocks monocrotalineinduced pulmonary arterial hypertension by improving inflammation and pulmonary artery remodeling. Int J Mol Med. 2021; 47 (2): 595-606.

3. Behringer A, Trappiel M, Berghausen EM, Ten Freyhaus H, Wellnhofer E, Odenthal M, Blaschke F, Er F, Gassanov N, Rosenkranz S, et al. Pioglitazone alleviates cardiac and vascular remodelling and improves survival in monocrotaline induced pulmonary arterial hypertension. Naunyn Schmiedebergs Arch Pharmacol. 2016; 389 (4): 369-79.

4. Chai Y, Zhu K, Li C, Wang X, Shen J, Yong F, Jia H. Dexmedetomidine alleviates cisplatininduced acute kidney injury by attenuating endoplasmic reticulum stressinduced apoptosis via the alpha2AR/PI3K/AKT pathway. Mol Med Rep. 2020; 21 (3): 1597-605.
